# Active membrane deformations of a minimal synthetic cell

**DOI:** 10.1038/s41567-025-02839-3

**Published:** 2025-03-24

**Authors:** Alfredo Sciortino, Hammad A. Faizi, Dmitry A. Fedosov, Layne Frechette, Petia M. Vlahovska, Gerhard Gompper, Andreas R. Bausch

**Affiliations:** 1 Lehrstuhl für Zellbiophysik (E27), Physik Department, Technische Universität München, Garching bei München, Germany; 2 Center for Protein Assemblies, Garching bei München, Germany; 3 Department of Mechanical Engineering, Northwestern University, Evanston, IL USA; 4 Theoretical Physics of Living Matter, Institute for Advanced Simulation, Forschungszentrum Jülich, Jülich, Germany; 5 Martin A. Fisher School of Physics, Brandeis University, Waltham, MA USA; 6 Department of Engineering Sciences and Applied Mathematics, Northwestern University, Evanston, IL USA

**Keywords:** Biological physics, Membrane biophysics

## Abstract

Living cells can adapt their shape in response to their environment, a process driven by the interaction between their flexible membrane and the activity of the underlying cytoskeleton. However, the precise physical mechanisms of this coupling remain unclear. Here we show how cytoskeletal forces acting on a biomimetic membrane affect its deformations. Using a minimal cell model that consists of an active network of microtubules and molecular motors encapsulated inside lipid vesicles, we observe large shape fluctuations and travelling membrane deformations. Quantitative analysis of membrane and microtubule dynamics demonstrates how active forces set the temporal scale of vesicle fluctuations, giving rise to fluctuation spectra that differ in both their spatial and temporal decays from their counterparts in thermal equilibrium. Using simulations, we extend the classical framework of membrane fluctuations to active cytoskeleton-driven vesicles, demonstrating how correlated activity governs membrane dynamics and the roles of confinement, membrane material properties and cytoskeletal forces. Our findings provide a quantitative foundation for understanding the shape-morphing abilities of living cells.

## Abstract (web summary)

How cells actively change their shape is an open question. Now, a reconstituted minimal cytoskeleton composed of microtubules and molecular motors is shown to produce membrane fluctuations that drive active shape changes in synthetic cells.

## Main

Rather than being merely passive containers, cell membranes actively respond to and steer cellular activity, enabling a myriad of biological functions such as cell crawling, cell division and cytoplasmic streaming^1–8^. Moreover, nucleus deformations can affect transcription^9^, and prebiotic membrane deformations might have influenced the origin of life^10^. To accomplish these activities, cells have the ability to dramatically change their shape, and many of these processes arise from a tight coupling between lipid membrane fluctuations (providing the necessary flexibility) and the underlying cytoskeleton, which provides the necessary active forces and directionality to induce deformations. The seminal discovery of ‘membrane flickering’ in red blood cells^11,12^ revealed how fluctuation analysis is essential to understand membrane-driven processes both in vivo^13–16^ and in vitro^17–19^. One general result at equilibrium is that the temporal relaxation of membrane fluctuations is tightly bound to its spatial correlations^20–23^, enabling the extraction of mechanical properties from both spatial and temporal measurements of membrane dynamics. However, in cells, cytoskeletal activity modifies both spatial and temporal behaviours of membrane deformations^13,14^, potentially breaking their interdependence dictated by thermodynamics^23–27^. Hence, in living systems, it is challenging to simultaneously measure the dynamics of both membrane and cytoskeleton imparting forces on it and to draw a precise link between cytoskeletal activity and its resulting fluctuations.

Giant unilamellar vesicles (GUVs) are a powerful tool to investigate, in a controlled minimal system, how membrane deformations behave^17,19,22^ and how they are affected by activity^28–35^. However, despite progress^32,36–47^, replicating cell-like shape deformations using a minimal system remains elusive. Here we address this issue by encapsulating a reconstituted cytoskeleton composed of an active microtubule (MT) network inside a deformable GUV, and analysing the resulting fluctuations and shape deformations.

## Vesicle shapes induced by active bundles

**Fig. 1:**
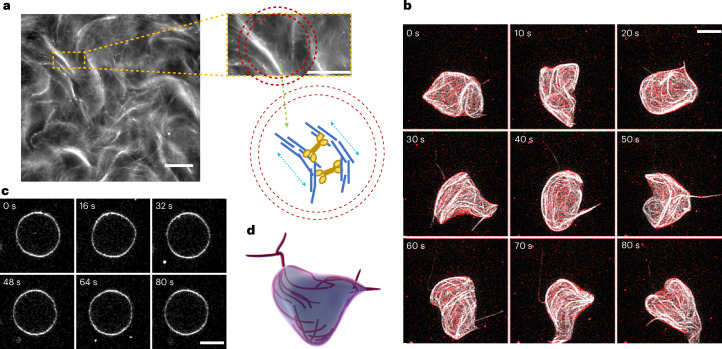
GUVs containing a minimal cytoskeleton exhibit large shape fluctuations. **a**, Confocal snapshot of the active MT network in unconfined conditions. Short fluorescently labelled MTs, in the presence of crosslinkers, self-organize into extensile active bundles, propelled by kinesin motors (right). Scale bar, 100 μm. This experiment was repeated twice. Top right: enlargement of the extensile bundles. A circle having a size comparable with the GUVs we produce (*R*_0_ ≈ 25 μm) is superimposed in red for reference. Scale bar, 50 μm. Bottom right: schematic of the encapsulated experimental system. Inside the GUV (red circle), small MTs of opposite polarities assemble into bundles that are extended (see arrows) by kinesin motors (in yellow). This results in active extensile bundles confined inside the GUV. **b**, Confocal projections of a GUV (membrane in red) containing an active MT network (MTs are shown in white). The GUV deforms and changes shape with a timescale of the order of several (~10) seconds. Scale bar, 20 μm. **c**, A passive vesicle fluctuating with the same time interval between frames as a reference. Scale bar, 20 μm. **d**, Artistic depiction of shape-morphing GUVs resulting from the encapsulation of active bundles.

Our experimental cytoskeleton model consists of an active gel of MTs and molecular motors, similar to previously reported systems^37,48–52^, encapsulated inside GUVs using the continuous droplet interface crossing encapsulation (cDICE) technique^53,54^. Microscopically, the active system is composed of short (~1 μm), stabilized MTs (at concentration *c*_MT_), kinesin tetramers (at concentration *c*_K_) and a crosslinker. Here, instead of the usual choice of having a depletant (such as polyethylene glycol), as a crosslinker, we use the protein anillin (at concentration *c*_A_), which induces filament–filament interactions leading to the formation of MT bundles (Supplementary Fig. 1a). Because of the additional activity of molecular motors acting on filaments, bundles extend and buckle due to kinesin-induced stresses (Fig. 1a), continuously breaking and aggregating, as long as adenosine triphosphate (ATP) is available (for 1 h, under our conditions). Using crosslinkers instead of depletants, and keeping the MT concentration in a dilute regime, we ensure that the bundles are not effectively attracted to the membrane (as they would in the presence of a depletion interaction) and are not dense enough to form a nematic material^55^. The net result is a minimal active cytoskeleton, which self-organizes into a loosely connected, isotropic three-dimensional network of long (~100 μm) extensile bundles. When encapsulated inside GUVs (mean radius, *R*_0_ ≈ 25 μm), this active MT network assembles inside the whole vesicle volume, in stark contrast to previous systems consisting of a dense two-dimensional nematic layer on the membrane^37^. Yet, when active bundles hit the membrane, the forces exerted by the MT gel induce large shape deformations of the vesicle (Fig. 1b and Supplementary Video 1). The GUV continuously undergoes dramatic morphological changes, with a timescale of ~10 s, as extracted by the correlation function of membrane deformations (Supplementary Fig. 5c). Active deformations are clearly different in magnitude and dynamics from those present at equilibrium (Fig. 1c). They are observed for a wide range of concentrations of MTs, kinesin and anillin (Supplementary Fig. 1b). These enhanced deformations are tightly correlated with the organization of the MT network inside the GUV (Fig. 1d and Supplementary Video 2). Since MTs do not assemble into a nematic layer, the deformations here are not localized to topological defects in the alignment of filaments^37^ but are a direct result of bundles exerting local forces on the membrane by their kinesin-induced extension. Moreover, vesicles never settle into a definite shape as observed in previous systems^28,30,32^, but rather continuously fluctuate around a spherical geometry.

## Fluctuation spectroscopy reveals enhanced out-of-equilibrium fluctuations

**Fig. 2:**
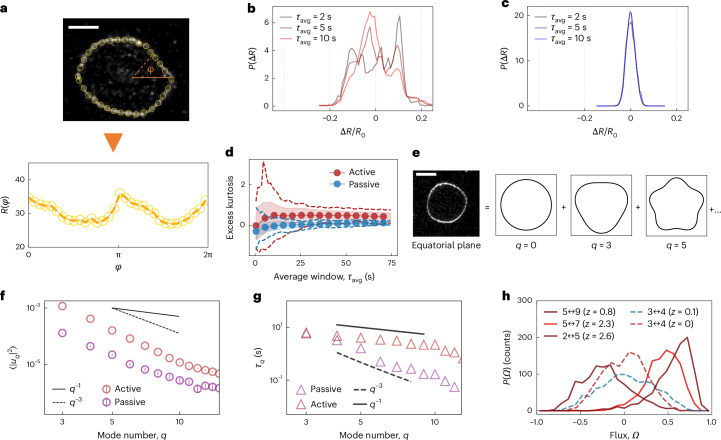
Membrane deformations of active vesicles are out of equilibrium. **a**, To analyse deformations, the equatorial contour of a fluctuating vesicle is tracked to obtain its description in polar coordinates *R*(*ϕ*, *t*). The extracted contour is marked by orange dots and then shown as a (*ϕ*, *R*) plot (bottom). The dashed line is a guide for the eye. Scale bar, 25 μm. **b**,**c**, Histograms of the distribution of radial deformations *P*(Δ*R*) for an active GUV (**b**), compared with a passive one (**c**), normalized by the mean radius *R*_0_. The contours are sampled for different times (*τ*_avg_ = 2 s, 5 s and 10 s) from the videos of GUVs. Active deformations have peculiar distributions at short timescales, indicating correlated dynamics. Passive GUVs exhibit a Gaussian-like distribution at all timescales, as expected from thermal noise. **d**, Excess kurtosis of the distribution of radial deformations as a function of sampling time *τ*_avg_ for an active (red) and a passive (blue) GUV. The circles indicate the mean, the shaded area indicates the s.d. and the dashed lines indicate the minimum/maximum kurtosis. Data are generated by extracting frames from recordings of a given duration *τ*_avg_ and computing the kurtosis of the distribution of radial deformations. At a short timescale, the difference in spread between active and passive is noticeable. **e**, Schematic of the decomposition of the contour into a sum of Fourier modes, labelled with *q*, whose fluctuations can then be separately analysed. **f**, Fluctuation spectrum for the Fourier coefficients *u*_*q*_ as a function of Fourier mode *q* for a passive (purple) and an active (red) GUV. Fluctuations of the active GUV are higher in magnitude and decay with a scaling exponent of ~3. The dashed line indicates a *q*^−3^ scaling and the solid black line, a *q*^−1^ scaling. Data are shown as mean ± standard error of the mean, derived from the precision of the contour detection based on the optical resolution of the camera. The error bars are smaller than the symbols. **g**, Correlation time *τ*_*q*_ at each mode *q* for a passive (purple) and an active (red) vesicle, as obtained from the correlation functions $\langle {u}_{q}^{* }(t){u}_{q}(0)\rangle$. Although the passive vesicle exhibits the expected *q*^−3^ scaling (dashed line), the active one has a different one (solid line indicates *q*^−1^). **h**, Histogram of the flux between mode *Ω* showing broken detailed balance between pairs of different modes, a signature of the out-of-equilibrium activity. The dashed blue line shows an example from a passive vesicle showing no net flux. For each dataset, we compute the *z* score as the mean divided by the s.d. (Supplementary Section 2.4), indicating how far the mean is from *Ω* = 0. One example in that even in the active case, there is no net flux (red dashed line).

More insights into the active membrane deformations can be gained by an analysis of the shape of the GUVs. First, we take long, high-frame-rate videos of the equatorial plane of a deforming GUV (30–4 frames per second; Supplementary Video 3) and extract its contour *R*(*ϕ*, *t*) (Fig. 2a). From this, we compute the distribution of membrane deformations Δ*R* = *R* – *R*_0_, where *R*_0_ is the mean radius. Deformations are averaged over a variable duration *τ*_avg_. We obtain large (~20%*R*_0_) deformations with a non-Gaussian distribution, especially for short *τ*_avg_ (Fig. 2b), indicating that the temporal behaviour of the active vesicles is distinct from the equilibrium counterpart (Fig. 2c), which instead shows smaller, Gaussian deformations. The difference is further validated by an analysis of the distributions’ kurtosis as *τ*_avg_ is varied, indicating that active GUVs at short times (*τ*_avg_ ≈ 2 s) show variations from a Gaussian distribution due to activity. At long averaging times instead (*τ*_avg_ ≈ 10 s), active deformations also approach bell-shaped distributions, although with a larger variance (Fig. 2d).

To gain further insights into the spatiotemporal dynamics of active GUVs, we resort to flicker spectroscopy. The contour is decomposed into Fourier modes, with mode number *q* (Fig. 2e and Supplementary Section 2) as

1 $$R(\phi ,t)={R}_{0}\left(1+\mathop{\sum }\limits_{q}^{{q}_{\max }}{u}_{q}(t){{\rm{e}}}^{{\rm{i}}q\phi }\right),$$

where *q*_max_ ≈ 15 is fixed by the image resolution. Each coefficient *u*_*q*_ represents the magnitude of equatorial deformations of wavelength ~*R*/*q*.

For passive vesicles with bending rigidity *κ* and tension *σ*, the power spectrum of the Fourier coefficients *u*_*q*_ is expected to scale as

2 $$\left\langle | {u}_{q}{| }^{2}\right\rangle \approx \frac{{k}_{{\rm{B}}}T}{\kappa }\frac{1}{{q}^{3}+\bar{\sigma }q},$$

where *k*_B_*T* is the thermal energy and $\bar{\sigma }=\sigma {R}_{0}^{2}/\kappa$ is the normalized tension. Note that this equation for the equatorial fluctuations of a GUV is the equivalent of the classical Canham–Helfrich expression but for a quasi-spherical membrane on an equatorially intersecting plane^17,20,21^. For passive vesicles, we recover the classical regimes of bending ($\bar{\sigma }\ll 1$; Fig. 2f, purple) and tension-dominated fluctuations ($\bar{\sigma }\gg 1$; Supplementary Fig. 2a). From these data, for passive GUVs made of egg phosphatidylcholine, we extract the bending rigidity of *κ*_pass_ = (13.4 ± 2.5) *k*_B_*T* (mean + s.d., *n* = 5). This value is consistent with those in the literature^34,56^, confirming that the cDICE approach to prepare GUVs does not affect their mechanical properties. Additionally, from vesicles in the tension-dominated regime (selected by their radius *R*_0_, which tunes the crossover mode $\bar{\sigma }$) and, hence, showing a clearer ~*q*^−1^ low-*q* decay, we also extract a passive membrane tension of *σ* ≈ 10^−7^ N m^–1^, to be considered as an estimate because tension will be different for each GUV and depend on their reduced volume. The spectra of passive vesicles encapsulating MT networks in the absence of ATP, or in the absence of motors and crosslinkers, resemble those observed for bare vesicles (Supplementary Fig. 2b).

By contrast, actively deforming GUVs exhibit fluctuations that are roughly one order of magnitude above the passive reference (Fig. 2f, red) at all mode numbers. From now on, all the spectra refer to the same vesicle with *c*_MT_ = 0.8 mg ml^–1^ MTs, *c*_A_ = 1.5 μM and *c*_K_ = 120 nM (further examples showing the effect of parameters variation are shown in Supplementary Fig. 2c and the reproducibility details are given in the Methods). The fluctuation spectra of the active GUVs decay over *q* similar to the observed passive bending-dominated case: 〈∣*u*_*q*_∣^2^〉 ≈ *q*^−3^. The increase in magnitude indicates that in the presence of activity, thermal excitation of the bending modes of the vesicle is negligible compared with the deformations driven by active forces^28,29,57^. The increase at a high mode number is in part due to the formation of transient tethers, which, however, are rare with respect to the overall smoother deformation.

We then turn to the temporal behaviour of deformations. The time correlation function $\langle {u}_{q}(\tau ){u}_{q}^{* }(0)\rangle$ of a purely passive GUV is expected to decay as an exponential^17^ with a decay time *τ*_*q*_, which, in turn, scales exactly like the spatial spectrum, as ${\tau }_{q} \approx {({q}^{3}+\bar{\sigma }q)}^{-1}$ (Fig. 2g). For active GUVs, however, we observe a *τ*_*q*_ ≈ 1/*q* scaling at low modes, which does not match the scaling of spatial fluctuations. This indicates that the activity strongly affects the timescales of membrane fluctuations and, thus, defies the relationship between fluctuations and response expected at equilibrium, imposing the same scaling between spatial fluctuations and their temporal decay. Indeed, using previously established methods^58,59^, we confirm that membrane fluctuations break detailed balance. Briefly, using the amplitude of Fourier modes as a proxy for the microscopic configuration of GUVs, we find the presence of net probability fluxes *Ω* in the transitions between different modes, which would be expected to vanish at equilibrium due to the detailed balance. Intriguingly, a statistically significant net flux is only present between some couples of modes, indicating that the active force couples preferentially with deformations of specific wavelengths (Fig. 2h and Supplementary Fig. 4). This confirms that the membrane is out of equilibrium and that its temporal dynamics can be informative about the microscopic details of activity-induced deformations.

## MTs set the correlation time of membrane fluctuations

Since the membrane fluctuations are induced by the contained minimal cytoskeleton, we then focus on the properties of MTs inside the GUV, taking advantage of our ability to image them at the same time as the membrane.

**Fig. 3:**
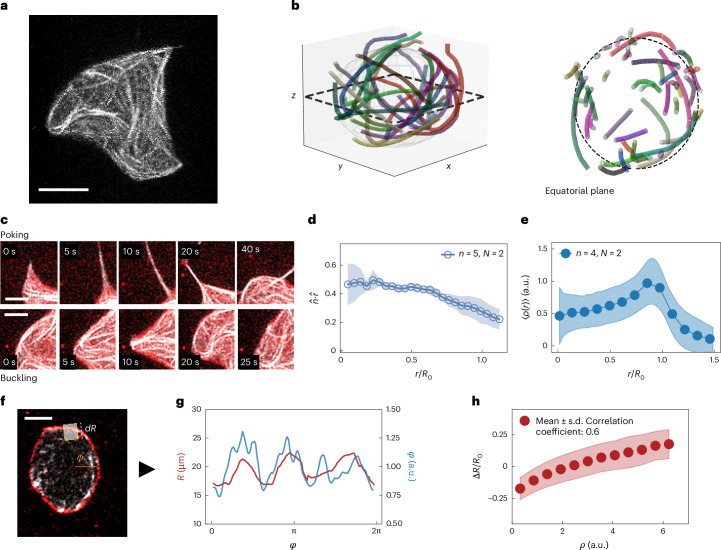
MTs act on the membrane to induce shape deformations. **a**, Confocal projection of the active MT network, showing how its structure is correlated with the GUV shape. Scale bar, 20 μm. Similar results have been obtained for GUVs in similar conditions (Methods). **b**, Tracking of MT bundles inside a GUV using SOAX, from 20 overlaid frames of a confocal recording. The GUV surface is filled with MT bundles (left), but bundles can also be present in unconfined conditions, as shown by a 5-μm-thick slice of the equatorial plane seen from the top (right). Only select bundles are shown, each with a different colour. The dashed line (left) indicates the equatorial plane selected on the right, and the dashed line (right) indicates the average GUV equatorial contour. **c**, Confocal time series, showing how MTs deform the GUV. Bundles can poke the membrane (top), leading to the formation of tubes, or they can (bottom) buckle against the membrane, inducing smoother shape deformations. MTs are marked in white and the membrane, in red. Scale bars, 5 μm. **d**, Close to the surface, MTs are mostly tangential to the membrane, as indicated by the small value of the dot product between the bundle orientation ($\hat{n}$) and radial vector ($\hat{r}$). The shaded area indicates the s.d. between *n* = 5 different GUVs analysed, from *N* = 2 experiments in the same conditions. **e**, Radial intensity profile of the MT density *ρ*(*r*), showing accumulation close to the membrane. The dashed line indicates the mean radius of the GUV over time. The shaded area indicates the s.d. between *n* = 5 different GUVs analysed, from *N* = 2 experiments in the same conditions. **f**, MT intensity *ρ*(*ϕ*, *t*) is obtained by averaging the MT fluorescence intensity in a box (coloured in yellow) centred at the membrane position *R*(*ϕ*, *t*) and with size *dR* = 5 μm (indicated by the white dashed line) to obtain the angular distribution of MTs along the membrane (not to scale in the picture). The angle *ϕ* is computed using the orange line as a reference (*ϕ* = 0). Scale bar, 20 μm. **g**, Plot of the MT density *ρ* (blue) and the local deformation Δ*R* (red) along the membrane, showing correlations between the two. A higher density leads to a higher deformation. **h**, Scatter plot of the correlation between the density and deformations along the whole trajectory of a GUV. Deformations are normalized by the mean radius *R*_0_ of the GUV. We find a Pearson correlation coefficient of 0.6, computed using Python’s numpy.corrcoef function. The shaded area indicates the s.d. between *n* = 5 different GUVs analysed, from *N* = 2 experiments in the same conditions.

With the bundle length (~100 μm) comparable with the GUV diameter (~50 μm), the active network affects the membrane fluctuations, whereas, in turn, the MT organization is altered by the confinement of the membrane. This membrane–cytoskeleton interaction leads to a complex, three-dimensional organization of the bundles (Fig. 3a and Supplementary Video 4). To better show their organization, we tracked bundles using the filament-tracking software SOAX^60^ and show that indeed different from previous systems encapsulating active MTs^37^, bundles are neither confined on the surface nor do they cover it completely (Fig. 3b).

We identify two main ways in which MTs can push against the membrane. When they extend and push radially against the membrane, they lead to transient, tube-like protrusions (‘poking’ behaviour; Fig. 3c (top) and Supplementary Video 5). Such tubes are transient and retract. Instead, when the bundles approach the membrane tangentially, they produce long-wavelength deformations, due to bundles buckling against the membrane (‘buckling’ behaviour; Fig. 3c (bottom) and Supplementary Video 6). In both cases, membrane deformations are closely correlated with the local activity of the MT network.

On the basis of these observations, we postulate that the temporal correlation of membrane deformations is connected to the dynamics of the MT bundles and under the assumption that the activity of filaments is proportional to their number, irrespective of their orientation, we propose to use the local MT density as a proxy for the force they exert. This assumption might be invalid if the filaments push radially and, hence, more effectively; however, we confirm that buckling is the main deformation mode by showing that the bundle orientation $\hat{n}$, extracted using SOAX, is tangential to the membrane when *r* ≈ *R*_0_ (Fig. 3d). From fluorescence videos, we then extract the intensity of MTs *ρ*(*r*, *ϕ*, *t*), where *r* is the distance from the centre of the GUV. We confirm again that filaments can be found in the GUV volume, but filaments are still more concentrated along the membrane surface due to their extensile behaviour; the radial density of MTs peaks at *r* ≈ *R*_0_ (Fig. 3e).

**Fig. 4:**
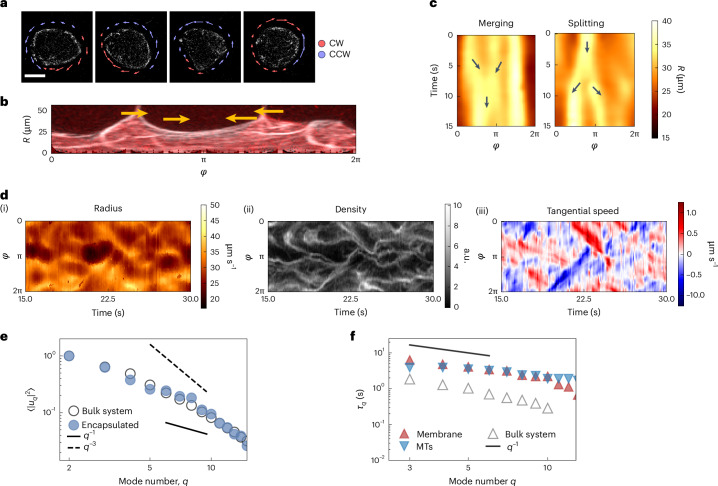
Dynamics of MTs sets the dynamics of membrane deformations. **a**, Arrows indicate the tangential component of the speed of MTs in the vicinity of the membrane at four different snapshots of a 6-min recording. The flow goes both clockwise (CW; red) and counterclockwise (CCW; blue), and is organized in domains of similar flow (transient waves) that move, collide and rearrange over time. The arrow colour only indicates the CW/CCW direction. Scale bar, 20 μm. **b**, By visualizing the vesicle using confocal images projected in the (*r*, *ϕ*) plane, where *r* is the distance from the GUV centre, one can see MT-driven membrane deformations that travel along the membrane, transported by the active flow (indicated by arrows). **c**, Kymographs of the membrane deformations *R*(*ϕ*, *t*) showing areas of high deformations propagating in time and merging (left) or splitting (right). The arrows indicate the direction of motion. **d**, Kymograph of membrane deformations (i), MT density (ii) and tangential flow along the membrane (iii), showing high correlation and indicating how flow transports the MT density, which, in turn, induces the membrane deformations. **e**, Spatial fluctuation spectrum for the MT density *ρ*(*ϕ*) both under GUV confinement but close to the membrane (full blue circles) and in unconfined conditions (open grey circles). Both spectra are normalized so that they have a value of 1 at *q* = 2. The two quantities show a similar decay. As shown in Fig. 2g, the dashed line indicates a *q*^−3^ scaling and the solid black line, a *q*^−1^ scaling. **f**, Correlation times *τ*_*q*_ of deformations at each mode *q* for the membrane (red) compared with those extracted from the spectral description of the MT density *ρ*(*ϕ*, *t*) (blue). The two curves are comparable with each other and display the same scaling. The same spectral analysis, performed on the unconfined system, shows a similar scaling but faster decorrelation. The solid black line shows a *q*^−1^ scaling.

Shape changes in the membrane are, hence, driven by the local organization and activity of the MTs, which directly push against the membrane and deform it. We can capture the relevant dynamics of filaments and their interplay with the membrane by reducing our observable to an angular density of MTs *ρ*(*ϕ*, *t*) ≔ *ρ*(*r* ≈ *R*_0_, *ϕ*, *t*) computed only in the vicinity of the membrane (Fig. 3f). By directly correlating the membrane deformations with the local MT density, we confirm that filaments are also highly concentrated in places where membrane deformations are larger than average (Fig. 3g–h). We then turn to the dynamics of MTs. By tracking the flow of MTs along the membrane (Supplementary Fig. 8a,b), we observed that clusters of highly concentrated MTs travel along the membrane with a speed of *v* ≈ 1 μm s^–1^ (Fig. 4a and Supplementary Videos 7 and 8), transporting with them the membrane deformations they induce (Fig. 4b). This gives rise to transient deformation waves that travel, merge or split (Fig. 4c and Supplementary Video 4), and then switch direction or dissolve after around 10 s. These transient waves are due to the tendency of the MT bundles to extend, thereby deforming the membrane. Collision with the membrane deflects the motion of MTs, thereby turning extensile activity into motion along the membrane. On average, the component of the velocity in the direction tangential to the membrane (*v*_T_) accounts for *v*_T_/*v* ≈ 80% of the total speed (Supplementary Fig. 8c). It follows that the dynamics of the membrane is due to MTs deforming it due to their extensile-based pushing and then moving along the GUV perimeter due to confinement. Consequently, they transport these deformations along the membrane, as confirmed by the correlation between radial deformations, MT density along the membrane and their tangential speed that propagates the deformations (Fig. 4d(i)–d(iii)).

To quantitatively understand the link between MTs and membrane fluctuations, we used again a Fourier series expansion of the MT density *ρ*(*ϕ*, *t*). The method mirrors the spectral analysis of membrane, being now focused on the active MT network pushing on the membrane. Briefly, we computed both density fluctuation spectrum 〈∣*ρ*_*q*_∣^2^〉 and its temporal decay for each mode. The fluctuation spectrum (Fig. 4e, blue) is distinct from that of the membrane. This aligns with what we see for an unconfined MT active gel (Supplementary Fig. 7, grey). This suggests that the angular filament density within GUVs is arranged similar to its distribution in bulk. This specific spatial arrangement of MTs, however, when acting on the membrane—although scaling differently than equatorial fluctuations—does influence them, resulting in the observed *q*-dependent decay of membrane fluctuations. In particular, conversely, the MT density also exhibits *q*-dependent decay times ${\tau }_{q}^{{\rm{MT}}}$, which are consistent in both magnitude and scaling with the decay times observed for membrane fluctuations (${\tau }_{q}^{{\rm{MT}}}\approx {\tau }_{q}$). This parallelism suggests that the active MT fluid determines the membrane fluctuation timescale (Fig. 4f and Supplementary Figs. 5 and 6). This elucidates the observed separation between the membrane’s spatial and temporal correlations, the latter being now solely set by the active MT network.

Moreover, again examining a similar MT system in bulk under identical conditions, we found a similar scaling of the correlation times but with faster decay times (around ten times shorter than inside GUVs). This indicates that the confinement induced by the GUV slows down the system dynamics (Fig. 4f, grey). Hence, soft confinement, although not modifying its angular distribution, extends the MT density’s correlation timescale. We attribute this effect to the membrane acting as a barrier, which redirects any radial flow of the network tangentially, thereby prolonging its temporal persistence.

## Theoretical coarse-grained model recapitulates the observed dynamics

**Fig. 5:**
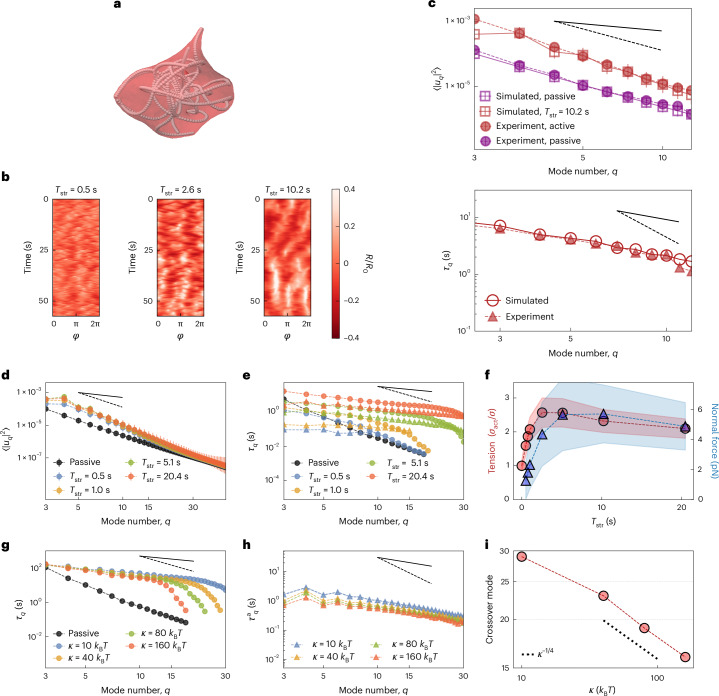
Simulations recover and extend the experimental results. **a**, Snapshot of numerical simulations, carried out for *T*_str_ = 10.2 s. Extensile filaments (white) are shown inside a simulated membrane (red) and deform it. **b**, Kymographs of equatorial membrane deformations (*R* is the distance from the GUV centre at a given angle *ϕ*) at different values of extension time *T*_str_, normalized by the mean radius *R*_0_. **c**, Top: spatial fluctuation spectrum of passive (purple) and active (red) membranes, from experiments (full circles) and simulations (empty squares; extension time, *T*_str_ = 10.2 s for the active case) showing similar scaling and magnitude. **c**, Bottom: simulated (empty circles) and experimental (full triangles) correlation times *τ*_*q*_ for each mode *q* for the active case, in the same conditions. In this and the subsequent plots, a dashed black line indicates an ~*q*^−3^ scaling and a solid black line, an ~*q*^−1^ one. The error estimate (mean ± s.d.) for simulations is explained in the Methods. **d**, Simulated spatial fluctuations at different values of *T*_str_, with passive reference in black, showing that the temporal dynamics of the force does not significantly affect the active spectrum. Error estimate (mean ± s.d.) for simulations is explained in the Methods. In **c** and **d**, the experimental and simulation errors are computed as explained in the Methods. **e**, Simulated temporal fluctuations of the membrane (*τ*_*q*_; circles) and of the active force (${\tau }_{q}^{{\rm{a}}}$; triangles) at different values of *T*_str_, with the passive reference (${\tau }_{q}^{{\rm{P}}}$) shown in black. Both force and membrane show the observed *q*^−1^ decay. **f**, Simulated tension *σ*_act_ and mean force acting on the membrane at different values of *T*_str_, showing a limited increase in both quantities. Mean ± s.d. are shown. **g**, Temporal fluctuations of the membrane at different values of *κ* with the passive reference, showing how rigidity affects only the low-*q* modes **h**, Simulated temporal fluctuations of the force (${\tau }_{q}^{{\rm{a}}}$) at different values of *κ*, with the passive reference shown in black. The bending rigidity *κ* does not significantly alter the temporal properties of the active force. **i**, Crossover mode at which the active temporal fluctuations stop scaling as *q*^−1^ as a function of *κ*. The dotted line indicates the scaling with the bending rigidity as *k*^−1/4^, compatible with the energy cost of bending a membrane.

To better understand the behaviour of the temporal and spatial fluctuations of active GUVs, we resort to the numerical simulations of a simplified model that nevertheless captures all the key characteristics of the experimental system. We implement a fluid membrane model based on dynamically triangulated surfaces^61,62^, consisting of a mesh of membrane points connected by bonds. The vesicle membrane has a diameter *D* = 40 μm, area and volume conservation are enforced (Supplementary Section 3), and we set the membrane bending rigidity and tension to *κ* = 20*k*_B_*T* and *σ* = 10^−8^ N m^–1^, respectively. Membrane hydrodynamics is ignored for simplicity, but this does not significantly affect the results (Supplementary Fig. 10e). The active cytoskeleton is implemented by a set of *N*_fil_ = 20 filaments placed inside the vesicle. Filaments have a relaxed length *L* = *D*/2 and can grow and extend for a typical time *T*_str_ to reach a maximum length *L*_max_ = 2*D*, before shrinking back. This growth dynamics simulates the extensile behaviour of MT bundles and their ability to buckle, whereas the shrinking simulates bundles breaking and/or leaving the membrane. Bundle activity leads to membrane deformations analogous to the experimentally observed ones (Supplementary Video 9 and Fig. 5a,b). Kymographs of the membrane deformations for different values of *T*_str_ show the ‘wave-like’ behaviour observed in experiments if *T*_str_ is high enough, indicating that filaments transport deformations in space by extending along the membrane. Conversely, for low *T*_str_, membrane deformations are only local and, as the filaments shrink, quickly relax with their passive decay time (Fig. 5b). We find good agreement between the simulated vesicles and the experimental measurements, both for passive and active GUVs and in both spatial and temporal decays of fluctuations (Fig. 5c). The simulated fluctuation spectrum is indeed roughly one order of magnitude higher than the passive reference and scales indicatively as ~*q*^−3^ (Fig. 5c, top), whereas the relaxation time of the membrane deviates from the equilibrium behaviour and shows a *q*^−1^ decay (Fig. 5c, bottom), in accordance with the experimental findings. We postulated that this behaviour of active vesicles arises whenever activity dominates the membrane dynamics, that is, if ${T}_{{\rm{str}}} > {\tau }_{q}^{{\rm{P}}}$, where ${\tau }_{q}^{{\rm{P}}}$ is the passive relaxation time of the membrane. Indeed, if ${T}_{{\rm{str}}}\ll {\tau }_{q}^{{\rm{P}}}$, the temporal relaxation is marginally affected, whereas for ${T}_{{\rm{str}}}\gg {\tau }_{q}^{{\rm{P}}}$, the membrane relaxation is fully driven by activity, exhibiting the peculiar *q*^−1^ scaling and overall increasing correlation time as *T*_str_ grows (Fig. 5e, circles). By measuring the correlation time at each mode of the active force ${\tau }_{q}^{{\rm{a}}}$ (Supplementary Section 2), we again show that the ~*q*^−1^ decay of the membrane correlation times is imposed by the temporal activity of the force (Fig. 5e, triangles). Interestingly, we observe this temporal scaling for different values of *N*_fil_, *L*_max_/*D* and the vesicle’s reduced volume (Supplementary Fig. 10). By contrast, the behaviour of the fluctuation spectrum is only mildly affected by all of the above parameters, indicating that the temporal behaviour of the membrane fluctuations, rather than its spatial counterpart, might be a better readout of the effect of activity. We then looked at a possible explanation for this behaviour. As ~*q*^−1^ decays are commonly associated with tension, we wondered whether the observed scaling could be explained in terms of an effective active tension *σ*_act_ by pushing forces^14,22^. We found that as *T*_str_ increases, the total tension indeed rises. At the same time, the mean active force acting on the membrane also increases (Fig. 5f). However, the effect is small, with only a moderate increase in tension due to the active force. We then tested the effect of membrane rigidity *κ*. As *κ* increases from 10*k*_B_*T* to 160*k*_B_*T* at fixed *T*_str_ = 10.2 s, the same ~*q*^−1^ scaling is observed, but only at lower modes, indicating an interplay between membrane mechanical properties and the active force (Fig. 5g). This effect, in which the membrane and the active force synchronize only at low modes, is not dependent on a change in the temporal correlation of the force, which is not modified by the bending rigidity (Fig. 5h) but depends only on the mechanical properties of the membrane. Indeed, the crossover mode at which the ~*q*^−1^ behaviour stops scales as ~*κ*^−1/4^, that is, the highest excitable mode is the one at which the force becomes comparable with the cost of bending deformations ~*κq*^4^ (Fig. 5i). Hence, an additional condition for the synchronization to happen is that the force is stronger than the membrane’s deformation cost. We conclude that this behaviour is universal, as we consistently observed an ~*q*^−1^ scaling in the membrane’s temporal relaxation as soon as activity dominates. The tight correlation between membrane deformation and force is confirmed in simulations, indicating that the behaviour of the membrane follows the behaviour of the filaments in the temporal domain. The observed fluctuations in the membrane and MT dynamics, hence, reflect a feedback in which soft confinement modifies the temporal MT organization, which, in turn, dictates large membrane fluctuations around the equilibrium spherical shape. The resulting activity of the MT network acting on the membrane is correlated in time and drives the dynamics of membrane fluctuations accordingly, as can be rationalized by the fact that the correlation function of a membrane under the effect of active correlated noise tends to synchronize with the slower timescale^13,14,24^, which is the active one here. Measuring both quantities directly allows to clearly prove it.

In summary, deformation dynamics are regulated by active forces, with temporal fluctuations better capturing out-of-equilibrium signatures than spatial ones^13,14,24,26,27^ and, hence, being a more direct observable of activity in living systems. Membrane deformations also allow to extract the typical time and force of the active system. Our system relies on a simplified cell and cytoskeleton model, whereas in cells, membrane deformations are rather a consequence of actin turnover dynamics. However, experiments recover typical timescales and mechanical properties of living cells. Additionally, GUVs also represent a model for protocells; hence, similar active fluctuations might shed light on early organizations of biomembranes. Although extreme, the deformations we observe do not lead to any transition from a spherical topology, which would be possible by including area growth, bringing it closer to origin-of-life conditions reported elsewhere^10^. Overall, understanding this dynamics is, therefore, a fundamental step in the programs of understanding biomembrane deformations and of reconstituting a realistic synthetic cell.

## Methods

### Buffers and proteins

M2B is 80 mM PIPES (pH 6.8), 2 mM MgCl_2_ and 1 mM EGTA. 3.2 mM MgCl_2_ is added at the end from a 67 stock already diluted in M2B.

Lipids (Texas Red DHPE and DOPC, Avanti Polar Lipids) are bought from Thermo Fisher. Anillin is purified in the laboratory from its sequence (Supplementary Section 1). Silicon and mineral oil are purchased from Sigma.

### Encapsulation using cDICE

Vesicles are produced using the cDICE method^38,53,54^ consisting briefly of letting droplets of the active mixture cross a layer of oil (silicon and mineral oil; Sigma) containing lipids, to coat them with a membrane. The droplets are produced by inserting a capillary in a three-dimensionally printed rotating chamber^38^. Finally, the vesicles accumulate in a buffered aqueous solution, whose osmolarity is matched to 20 mOsm kg^–1^ higher than the active mix using glucose. The mixture is prepared as follows: the desired concentration of stabilized MTs is mixed in M2B together with the desired concentration of kinesins, anillin and 2 mM ATP. The mixture also contains a scavenging system (10 U ml^–1^ glucose oxidase and 1 kU ml^–1^ catalase; Sigma), glucose (3 mg ml^–1^), an ATP regeneration system (18.2 U ml^–1^ of creatine phosphokinase and 9 mM of creatine phosphate; Sigma) and 5 mM dithiothreitol. It is ensured that the final concentration of salts is exactly the one expected for M2B by correcting using a 10× M2B preparation. The stock of MTs and the mixture are kept at room temperature to avoid depolymerization. After mixing, we wait for 5 min for the active fluid to assemble. Its osmotic pressure is measured using a Gondotec osmometer, and a buffered solution, containing glucose, with comparable osmotic pressure is also prepared. Finally, the cDICE encapsulation is carried out at room temperature. We use a capillary with a diameter of 40 μm to allow for big vesicles and fast encapsulation (5 min). The vesicles are then harvested, transferred to a glass coverslip coated with 1 mg ml^–1^ bovine serum albumin (Sigma) and observed under a microscope.

### Imaging

Vesicles are acquired using a confocal microscope (Leica DMi5, ×63 objective, 1.4 numerical aperture; LAS X, acquisition software) equipped with a resonant scanner. Long time series of the equatorial plane are acquired using a 256 × 256 image size with a time interval of 35–250 ms (around 4,000 frames, for a total recording time of 5–10 min per GUV). The pinhole was set to 1 Airy unit. Both membrane and MT channel are acquired at the same time. Three-dimensional stacks are also acquired at different resolutions and time intervals.

### Extraction of GUV contour and MT density

The images of the membrane channel are thresholded and the GUV contour is obtained using custom-written Python 3, MATLAB (version R2024) and Mathematica codes. Briefly, using the centre of the GUV as a reference, the space is divided in overlapping angular segments of amplitude *da* = 0.25 rad and separated by *dϕ* = 0.15, inside which the radial position of the point with the maximum intensity of the membrane is found, thereby obtaining a discretization of *R*(*ϕ*). The same logic is applied to the MT intensity to obtain *ρ*(*ϕ*, *r*), which can then be averaged inside a box of width *da* and contained between *R*(*ϕ*) and *R*(*ϕ*) – *dR* with *dR* = 5 μm to obtain *ρ*(*ϕ*) in proximity of the membrane.

### Kurtosis analysis

The extracted contours are split in a series of lengths *τ*_avg_; from each series, the histogram of the deformations (*R* – *R*_0_)/*R*_0_ (where *R*_0_ is the mean radius of the GUV over time) is extracted. The kurtosis for each resulting distribution is then computed and normalized so that the expected value for a Gaussian is 0 (excess kurtosis). For each value of *τ*_avg_, we then obtain a list of kurtosis collected and from which their average, standard deviation (s.d.), and minimum and maximum values are computed.

### Fluctuation spectra and decay times

The discretized version of *R*(*ϕ*) and *ρ*(*ϕ*) are, at each time point, expanded in a Fourier series to obtain the complex coefficients *u*_*q*_(*t*). For this, we compute the integrals *a*_*q*_(*t*) = (1/π)∫*R*(*ϕ*, *t*)sin(*qϕ*)d*ϕ* and *b*_*q*_(*t*) = (1/π)∫*R*(*ϕ*, *t*)cos(*qϕ*)d*ϕ*, using the trapezoidal rule. From this, we obtain *u*_*q*_(*t*) = (–*a*_*q*_(*t*), *b*_*q*_(*t*)). Alternatively, *R*(*ϕ*, *t*) is Fourier transformed with a fast Fourier transform to obtain the complex coefficients *u*_*q*_(*t*), appropriately scaled to match the mode number *q*. The variance of *u*_*q*_(*t*) yields the spatial spectrum, whereas its correlation function is used to extract the decay times. The decay time is defined as the time at which the correlation decreases below 1/*e* of the initial value to compare decays that are not strictly speaking exponentials.

### Statistics and reproducibility

Given the complexity and the large variation between vesicles, data are not pooled together, and only one GUV is shown in the text. The error bars on the individual spectra are obtained using the standard error of the mean from contour detection, based on the optical resolution of the camera. Other GUVs in the same conditions shown in the main text are discussed in Supplementary Figs. 2 and 3, including data on the GUV-to-GUV and day-to-day reproducibility of the experiment. Vesicles in different conditions are shown in Supplementary Fig. 1. Qualitatively, all the observed GUVs show the same trends, that is, an enhanced fluctuation spectrum and a synchronization between membrane and MT density in time. GUVs containing the active fluid but not showing active behaviour were excluded from the analysis and attributed to a faulty encapsulation process. No randomization or blinding was used. Errors in the simulation are computed as the mean ± s.d. of the quantities over 16,500 snapshots.

### Broken detailed balance

We extract the microscopic configurations of GUVs by decomposing their contours into different Fourier modes. The resulting (discretized) Fourier coefficients act as a proxy for a given configuration, and the probability of a given configuration is defined as the ratio of the time spent with a given set of Fourier coefficients over the toal time of the acquisition. The currents across the box boundaries determined by counting statistics, that is, the transitions between boxes yields the the probability current **j**. A non-zero value of its contour integral $\varOmega =\frac{{\oint }_{{{C}}}{\bf{j}}\cdot {\rm{d}}{\bf{l}}}{{\oint }_{{{C}}}|\,{\bf{j}}| \,{\rm{d}}l}$ indicates a system out of equilibrium, where *C* is a cycle. The flux is normalized and, hence, dimensionless. More details about this method are provided elsewhere^58,59^. The *z* score of each flux is obtained by collecting all the values of *Ω* across different cycles, taking their average and checking how many s.d. values away from *Ω* = 0 it is.

### Bundle tracking

Bundles are tracked using SOAX^60^ based on active contours. Confocal stacks of active GUVs are analysed, and the bundle contour segmented in positions **r**_*i*_ is extracted and plotted in three dimensions. Their local orientation is given by **n**_*i*_ = **r**(*i* + 1) – **r**_*i*_, enabling to find their alignment with respect to the radial direction. As **n**_*i*_ and –**n**_*i*_ are equivalent, the alignment is chosen to have a positive radial component.

### Analysis of MT flow

To extract the flow of MTs inside GUVs, the videos of the MT channel at the equatorial plane are analysed using an optical flow algorithm using a custom Python 3 script. Roughly, the intensity is followed over time extracting its flow, and then, the flow close to the membrane (using the procedure detailed above for the MT density) is averaged over 1 s and decomposed into tangential and radial components by a scalar product with a unit vector starting from the centre of the GUV and extending radially and its normal counterpart (tangential).

### Bulk experiments

To perform bulk (unconfined) experiments, the same mixture is injected inside a 10-μl microscopy chamber composed of a glass slide and a coverslip separated by a layer of parafilm. The slides and coverslips are passivated using polyacrylamide^52^. The Fourier analysis of the unconfined fluid is detailed in Supplementary Section 2.6.

### Coarse-grained modelling

In simulations, the membrane is modelled as a two-dimensional dynamically triangulated network, in which bonds between neighbouring vertices can be cut, flipped and reattached to mimic the internal reorganization of a fluid lipid bilayer. The extensile MT bundles are described by linear bead-spring chains with bending rigidity. The extension/retraction is described by a linear temporal growth of the bond lengths. The interaction of the filaments with the membrane is taken to be purely repulsive. Details and parameters are given in Supplementary Section 3.

### Reporting summary

Further information on research design is available in the Nature Portfolio Reporting Summary linked to this article.

## Online content

Any methods, additional references, Nature Portfolio reporting summaries, source data, extended data, supplementary information, acknowledgements, peer review information; details of author contributions and competing interests; and statements of data and code availability are available at 10.1038/s41567-025-02839-3.

## Supplementary information

Supplementary Information Supplementary Sections 1–3, Figs. 1–12, Table 1, Methods and References.

Reporting Summary

Supplementary Video 1 Confocal projection of a vesicle containing an active MT network and undergoing shape changes. The vesicle contains 0.8-mg ml^–1^ MTs, 120-nM kinesin and 1.5-μM anillin. MTs are shown in white and the membrane, in red. Time interval is 2.5 s. Scale bar, 50 μm.

Supplementary Video 2 Confocal three-dimensional reconstruction of a vesicle containing an active MT network. The vesicle contains 0.8-mg ml^–1^ MTs, 120-nM kinesin and 1.5-μM anillin. MTs are shown in white and the membrane, in red. Time interval is 2.5 s. Scale bar, 50 μm.

Supplementary Video 3 Equatorial fluctuations of an active vesicle. MTs are shown in white and the membrane, in red. Time interval is 100 ms. Scale bar, 50 μm.

Supplementary Video 4 Confocal three-dimensional reconstruction of the active MT network inside an active vesicle. The left pane shows the *z* projection over time, whereas the two other panes show a three-dimensional reconstruction from two different points of view. The vesicle contains 0.8-mg ml^–1^ MTs, 120-nM kinesin and 1.5-μM anillin. Time interval is 2.5 s. Scale bar, 50 μm.

Supplementary Video 5 Details of a vesicle undergoing poking. The vesicle contains 0.8-mg ml^–1^ MTs, 120-nM kinesin and 1.5-μM anillin. MTs are shown in white and the membrane, in red. Time interval is 2 s.

Supplementary Video 6 Details of a vesicle undergoing buckling. The vesicle contains 0.8-mg ml^–1^ MTs, 120-nM kinesin and 1.5-μM anillin. MTs are shown in white and the membrane, in red. Time interval is 2 s.

Supplementary Video 7 Flow of MTs in the proximity of the membrane inside an active vesicle. The blue arrows indicate counterclockwise flow, and the red arrows indicate clockwise flow. The velocity is extracted from the data using the optical flow of MT channel and computed in a 2-μm-wide area in proximity of the membrane. The arrow size indicates the magnitude of the speed. MTs are shown in white. Time interval is 4 s. Scale bar, 20 μm.

Supplementary Video 8 A fluctuating vesicle is projected in the (*R*, *ϕ*) plane to highlight the motion of membrane fluctuations along the membrane. Time interval is 2.5 s.

Supplementary Video 9 Simulations of active vesicles, with the membrane shown in red and active filaments, in white. Two different GUVs are shown (*T*_str_ = 1 s, left; *T*_str_ = 10.2 s, right).

## Data Availability

All raw data used in the Article are available via Zenodo (10.5281/zenodo.11351857)^63^, which includes source data for the images and plots in the main text.

## Code availability

Scripts used for the analysis of the data are available via Zenodo (10.5281/zenodo.11351857)^63^. The membrane model used for simulating the membrane is available from the corresponding author G.G. upon reasonable request.
