# Supplementary material for: Active membrane deformations of a minimal synthetic cell
Source: Nat Phys. 2025 Mar 24;21(5):799–807. doi: 10.1038/s41567-025-02839-3 (PMC12084157; doi:10.1038/s41567-025-02839-3)
Supplement: Supplementary file 1 — Supplementary Sections 1–3, Figs. 1–12, Table 1, Methods and References. [file 41567_2025_2839_MOESM1_ESM.pdf]

nature physics

Supplementary information https://doi.org/10.1038/s41567-025-02839-3

Active membrane deformations of a minimal synthetic cell

In the format provided by the authors and unedited

Contents

1 Methods and Material **2**
1.1 Anillin purification . . . 2

2 Fluctuation spectroscopy and data analysis **2**
2.1 Extraction of GUV's contour . . . 2
2.2 Growing semiflexible filaments . . . 4
2.3 Fluctuation spectra and decay times . . . 4
2.4 Broken Detailed Balance . . . 5
2.5 Analysis of the angular density of microtubules . . . 6
2.6 Analysis of bulk fluid . . . 6

3 Dynamically triangulated membrane model of a fluid vesicle **7**
3.1 Dissipative particle dynamics method for fluid modeling . . . 11
3.2 Growing semiflexible filaments . . . 12
3.3 Simulation setup and extracted quantities . . . 14
3.4 Computation of spectra and correlation times . . . 15
3.5 Computation of the active force and the corresponding τ_q^a values . . . 16
3.6 Computation of the tension . . . 16
3.7 Computation of the crossover mode . . . 16

1 Methods and Material

1.1 Anillin purification

Anillin (ANLN) is an actin-binding protein that bundles filaments. We use it as an (unspecific) microtubule crosslinker. We clone ANLN using the following (His-Tag containing) amino acid sequence:

MGSSHHHHHHSSGLVPRGSHMDPFTEKLLERTRARRENLQKKMADRPTAGTRTAAL
NKRPREPLLEANHQPPAPAEEAKPSSKPSPSKRRCSDNASTPDAGAENKQPKTPEL
PKTELSAVASHQQLRATNQTPQVSLLSSDKELTASDVKDASSVKTRMQKLADQRRY
WDNNVSPSSSPPAHVPPKDIIVSPPKPQIPDVGNDTPVGRRGRFANLAATIGSWED
DLSHPFVKPNNKQEKPGTACLSKESTTSSASASMNSRSVKQDTTSCSQRPKDTTVN
KAVCSGQLKNILPASKPASSVASTEVSGKSKPLAIKSPTVVTSKPNENVLPASSSL
KPVSANSSPQKTERPASRIYSYQSASARNELNNNTPVQTQQKDKVATSGGVGIKSF
LERFGEKCQEHSPAPLNQGHRTAVLTPNTKSIQERLLKQNDISSTALEHHHHHH

We use a pET28b(+) vector. Plasmids are amplified with chemically competent E. coli XL-1 blue cells for DNA production and E. coli BL-21 CodonPlus for protein expression, both purchased from Agilent Technologies.

Experimental methods

2 Fluctuation spectroscopy and data analysis

2.1 Extraction of GUV's contour

We acquire movies of the equatorial plane of fluctuating vesicles, starting from the membrane channel. The procedure is the same for active or passive vesicles. To extract the contour, we use previously developed algorithms [1]. The program was coded and compiled on MATLAB (Mathworks, USA) with the help of in-built MATLAB functions or using Python3 scripts. The software performs three important steps to detect the contour; (i) image processing, (ii) pixel and sub-pixel resolution contour detection and (iii) fitting the vesicle contour by a Fourier series. The details are as follows:

After acquiring the raw confocal fluorescence images of the membrane, we implement an inbuilt MATLAB sobel disk filter $fspecial$ $('sobel')$ and image normalization to increase the contrast of the contour. Initially, an estimated radius is determined by converting 10-100 consecutive contours into binary images. A skeletonization (*bwmorph*) and circle fitting algorithm (least square method) is used to determine an approximate radius of the vesicle. The first approximated radius is used to implement an inbuilt MATLAB function $imfindcircles()$. The function uses circular Hough transform to give an improved approximated radius R_{ves} and first guess of the centroid of the circle. This forms our preliminary image detection technique. The analysis becomes more precise by using the approximated centroid and R_{ves}. The cartesian plane in the MATLAB grid is converted to polar grid where the centroid is placed at the origin. The software proceeds to find the pixel location of minimum intensity in N regions of size $d\phi = 2\pi/N$ (wedges). It is to be noted that N is determined in powers of 2 to implement the Discrete Fast Fourier Transform (FFT) algorithm. The new centroid is determined by averaging the coordinates of the minimum pixel location. The improved approximated radius is found by determining the mean of each found pixel location from the new centroid. This

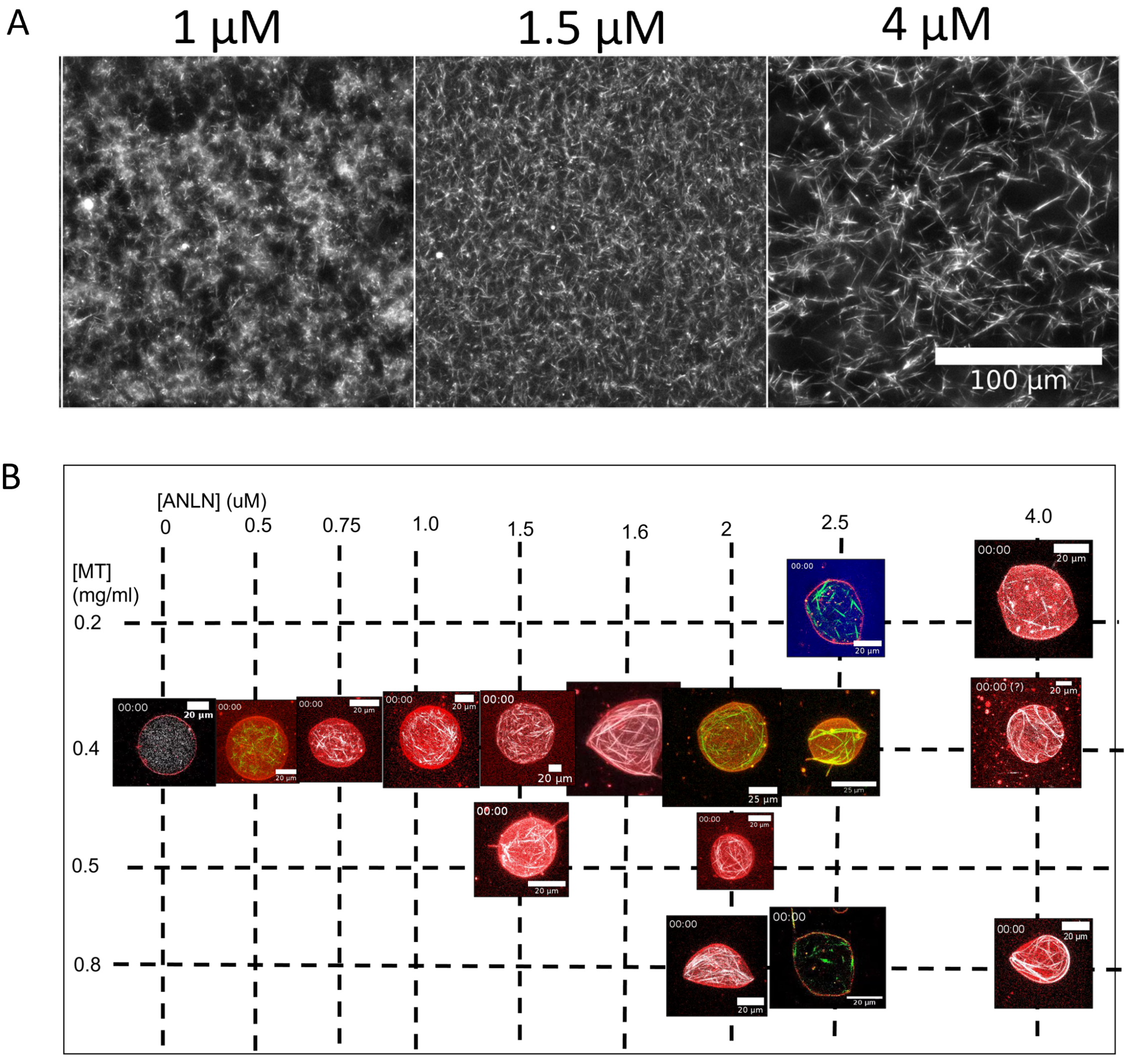

Supp.Fig. 1: A)Anillin bundles microtubules. Adding different concentrations of anillin to a mixture of 8 mg/ml of microtubules leads to the formation of bundles. Each conditions has been repeated twice. B) Active vesicles are observed for a range of ANLN and MT concentration. All GUVs contain 60 nM Kinesin in addition to the indicated concentrations.

process is iterated until the centroid point converges with a difference 0.001%. It is important to note that the contour is contained within an annulus (Region of Interest) of inner radius R_i and outer radius R_o; R_i<R_{ves}<R_o. This optimizes the software to work faster and prevents the algorithm from finding some defects or impurities in the images as the pixel location of the minimum intensity. After getting a converged centroid, the same algorithm of finding pixel location of minimum intensity is utilized. This is the gross signal of the contour with a pixel resolution accuracy. For the sub-pixel accuracy detection, we have implemented the algorithm from Gracia et al [2]. In summary, the sub-

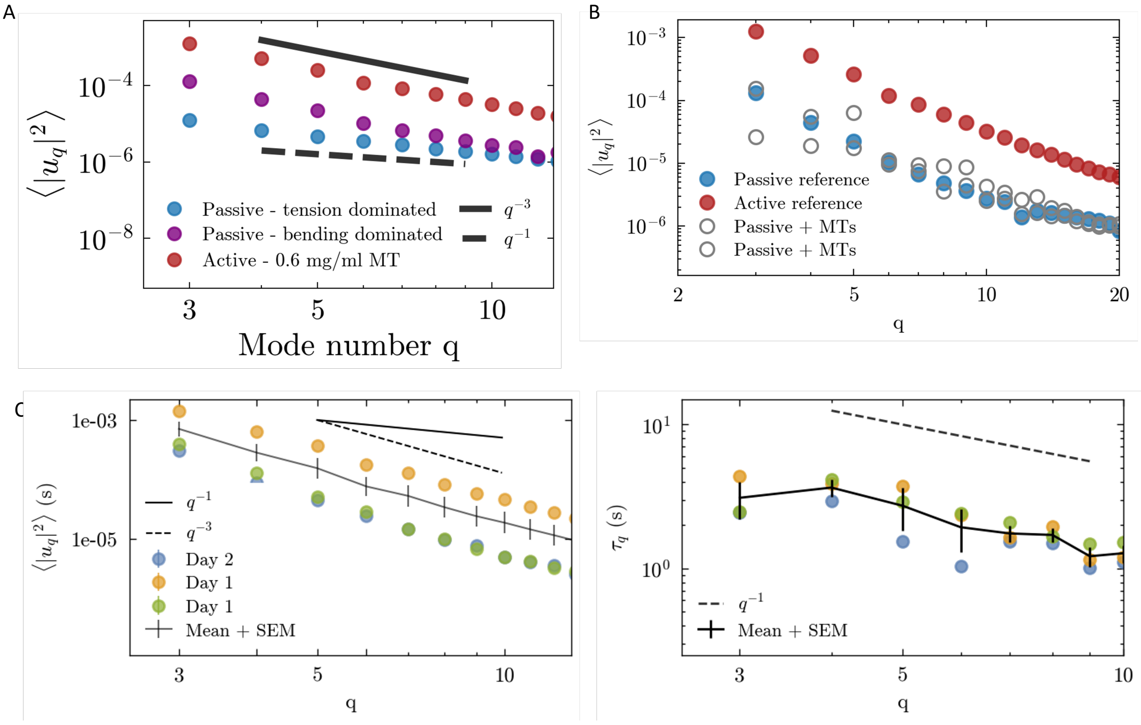

Supp.Fig. 2: A) Fluctuation spectra of an active and two passive GUVs (ref Fig. 2 of main text). The blue dots indicate a tension-dominated GUV with the spectrum scaling as $\sim 1/q$ obtained by choosing bigger GUVs so that the crossover mode between bending and tension-dominated regime is observable. B) Fluctuation spectra for two passive vesicles encapsulating microtubules but without crosslinkers and motors (open gray circles), showing that passive, un-bundled microtubules do not significantly modify the fluctuations. An empty passive vesicle (blue) and an active (red) spectra are shown as references. C-D) Fluctuation spectra (C) and correlation times (D) of three different active GUVs, showing comparable scaling and timescale.

pixel algorithm works as follows: we perform a fitting of the gray value intensity profile around the gross location of the contour points. This yields sub-pixel accuracy of the Fourier signal. The pixel accuracy algorithm detects the minimum point of the valley of the profile. The sub-pixel resolution contour detection is determined by fitting two slope lines around the minimum intensity pixel.

2.2 Growing semiflexible filaments

2.3 Fluctuation spectra and decay times

To perform flickering spectroscopy, we use a previously developed method as detailed in [1]. In summary, a time series of fluctuating vesicles at the equatorial cross section is recorded. The fluctuating contour is represented in Fourier modes, $r(\phi) = R\left(1 + \sum_q u_q(t)\exp(\mathrm{i}q\phi)\right)$ using a Fast Fourier Transform or explicitly performing the discrete integrals $a_q(t) = (1/\pi)\int R(\phi,t)\sin(q\phi)d\phi$ and $b_q(t) = (1/\pi)\int R(\phi,t)\cos(q\phi)d\phi$, using the trapezoidal rule (*trapz()* from the Numpy library of Python3) from which we obtain $u_q = (-a_q, b_q)$.

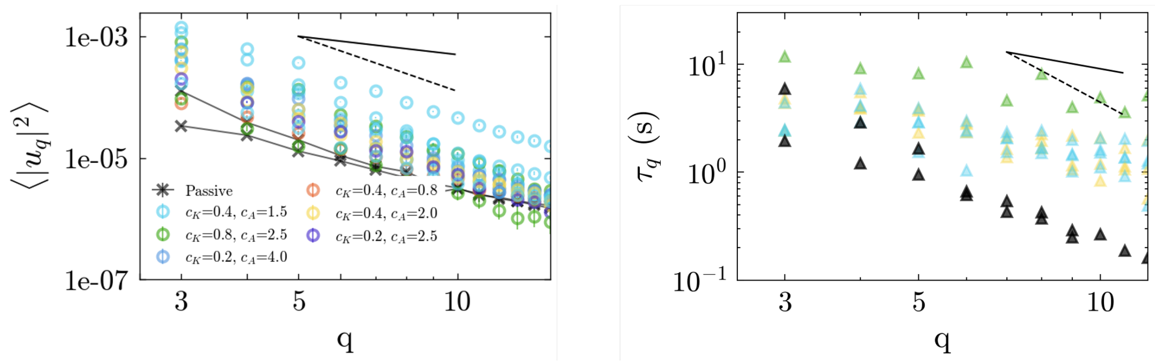

Supp.Fig. 3: Left) Fluctuation spectra in different conditions including a change in crosslinkers (c_A = 2.5 μM) with respect of the GUV shown in main text (c_A = 1.5; μM, c_K = 120 nM, c_{MT} =0.8 mg/ml), a variation in the number of microtubules (c_{MT} = 0.4 mg/ml). In all cases the ratio of MTs to motors is kept fixed (0.8 mg/ml:120 nM). Most spectra collapse on a $\sim q^{-3}$ curve roughly one order of magnitude above the passive reference (black). Right) Correlation times for the same vesicles. All active GUVs exhibit a similar $\sim q^{-1}$ scaling despite parameter variations. Colors are the same as in left plot, passive is shown in black.

The amplitude of the fluctuations u_q can be presented with mean square amplitude that depends on the membrane bending rigidity κ and the tension σ, $\langle|u_q|^2\rangle \sim \frac{k_BT}{\kappa(q^3+\bar{\sigma}q)}$, where k_BT is the thermal energy (k_B is the Boltzmann constant and T is the temperature), and $\bar{\sigma} = \sigma R^2/\kappa$. Images are acquired with confocal microscopy at 10-30 fps for 5-10 mins. Only vesicles with low tension value ranging from $10^{-7} - 10^{-10}$ N/m are chosen. This results in a small cross over mode given by $q_c = \sqrt{\bar{\sigma}}$ where the shape fluctuation modes are dominated by bending rigidity. We have ignored the ellipsoidal mode (q =2) as it is weighted with most excess area which leads to fluctuations with an increased amplitude.

Using the same time series data, one can also compute the temporal autocorrelation function which gives information about time evolution of the modes, $\langle u_q(0)u_q^*(t)\rangle = \langle|u_q|^2\rangle \exp(-t/\tau_q)$. If $q \gg 1$, the correlation time tends to that of a planar membrane $\tau_q^{-1} = \kappa\left(q^3 + \bar{\sigma}q\right)/4R_0^3\eta$. For tensionless vesicles, the correlation time would scale as $\tau_q^{-1} \sim \kappa q^3/4R_0^3\eta$.

2.4 Broken Detailed Balance

The non-equilibrium nature of the vesicle fluctuations can be quantified by the method of broken detailed balance. The method states that for a system in equilibrium, driven by thermal forces, observable microscopic configurations must be pairwise balanced. This refers to equal likelihood for the forward and backward transition between two processes. In our case, the microscopic configurations refer to the shapes defined by different Fourier modes. A non-equilibrium system, however, would display a probability flux cycle in the phase space of shapes; this refers to unequal rate of transitions between forward and backward shape changes. On the other hand, the probability is defined as the fraction of the time spent at a given shape configuration. A nonzero value of the contour integral of the probability current, $\Omega = \frac{\oint_C \mathbf{j}\cdot d\mathbf{l}}{\oint_C |\mathbf{j}|\ dl}$, indicates a system out of equilibrium. More details about the method can be found in [3, 4].

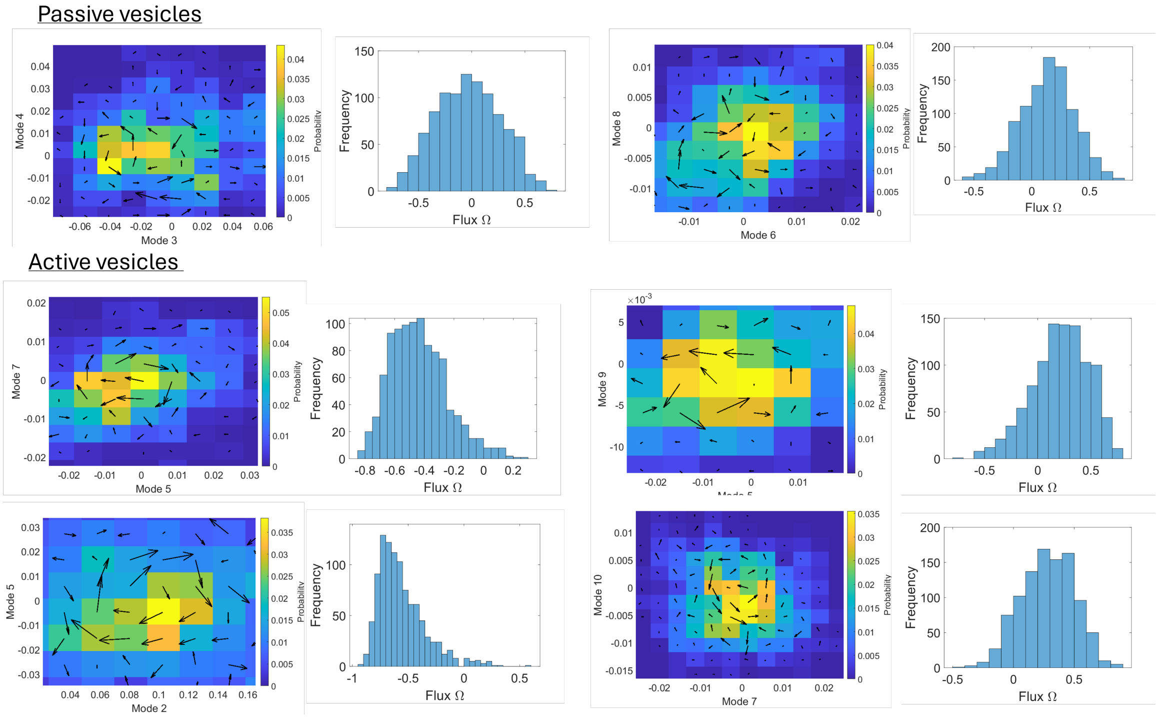

Supp.Fig. 4: Map and histogram of the flux Ω between different modes for passive vesicles (top) and active ones (bottom). Only active ones show overall net flux between modes. The maps illustrates the probability density between the two chosen Fourier modes. Colors indicate the probability of a given point in phase space (u_i, u_j), the size of the arrows indicate the currents across box boundaries determined by counting statistics - that is, the transitions between boxes.

2.5 Analysis of the angular density of microtubules

From the position of the membrane $R(\phi, t)$ we compute the angular density of microtubules close to the membrane $\rho(\phi, t)$ by averaging the microtubule fluorescence intensity inside a box of size 2 μm centered at $R(\phi, t)$. The density is normalized by the mean intensity over all boxes. The spacing between boxes is the same as the angular resolution $d\phi$ used to compute the membrane position.

2.6 Analysis of bulk fluid

To analyse the bulk fluid and compare it to the GUV, starting from time-lapse movies of the bulk fluid the angular intensity of microtubules is computed by measuring the fluorescence intensity inside virtual circles of radius ≈ 25 μm and thickness $dR = 2$ μm. The virtual circles mimic the procedure based on the GUV membrane as above. Having acquired the angular density $\rho^B(\phi, t)$, it is decomposed in Fourier modes as above and the spectrum and correlation times are computed (Fig. S7).

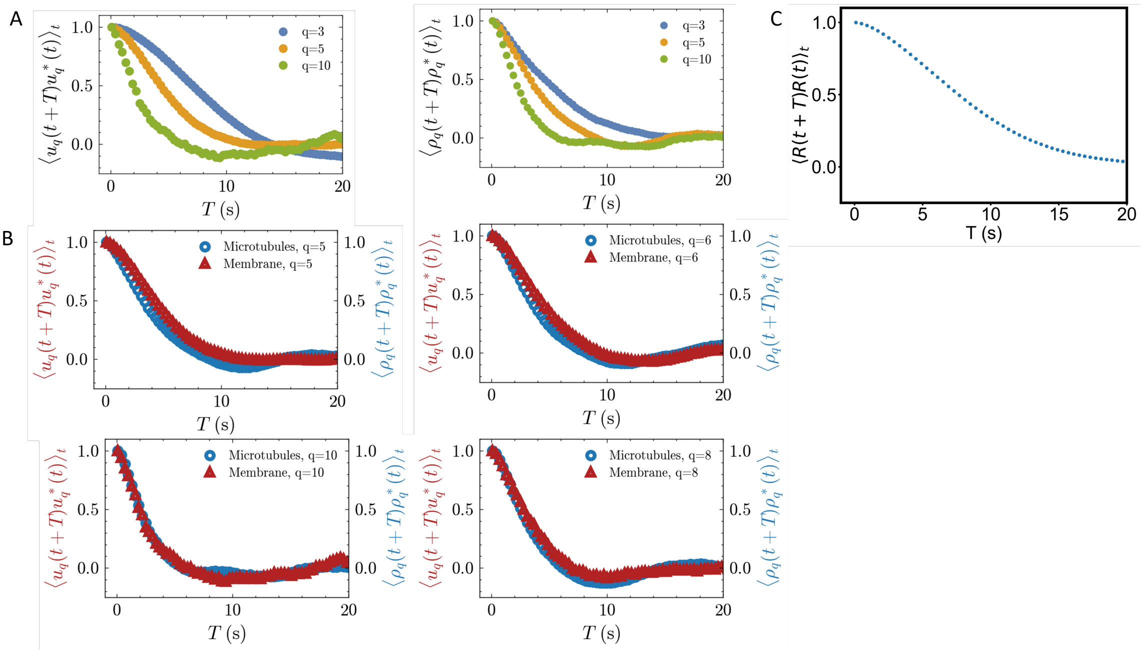

Supp.Fig. 5: A) Correlation function over time for modes $q = 3,\ 5,\ 10$ of both the membrane coefficients u_q (left) and the microtubules density ρ_q (right) showing they decay similarly and they have a roughly exponential decay especially at higher modes. B) Comparison for different values of q between the membrane (red) and the density (blue) correlation functions, showing that in the presence of activity both quantities decay in the same way. C) Correlation function in real space of the radial deformations, showing the radial deformations are correlated for ≈ 5 seconds in real space.

Simulations

3 Dynamically triangulated membrane model of a fluid vesicle

A lipid membrane is modeled by a dynamically triangulated network of N_{v} linked vertices [5, 6]. The links are represented by a tethering potential [7, 6] as

$$U_{\mathrm{att}}(r) = \begin{cases} k_{\mathrm{b}} \frac{\exp\left[1/(l_{c_0}-r)\right]}{l_{\max}-r} & \text{if } r > l_{c_0} \\ 0 & \text{if } r \le l_{c_0} \end{cases}, \tag{1}$$

$$U_{\mathrm{rep}}(r) = \begin{cases} k_{\mathrm{b}} \frac{\exp\left[1/(r-l_{c_1})\right]}{r-l_{\min}} & \text{if } r < l_{c_1} \\ 0 & \text{if } r \ge l_{c_1} \end{cases}, \tag{2}$$

where k_{b} is the bond stiffness, $l_{\min}$ and $l_{\max}$ are the minimum and maximum bond lengths, and l_{c_1} and l_{c_0} are the potential cutoff lengths. Thus, membrane vertices can move freely in the range $[l_{c_1}, l_{c_0}]$.

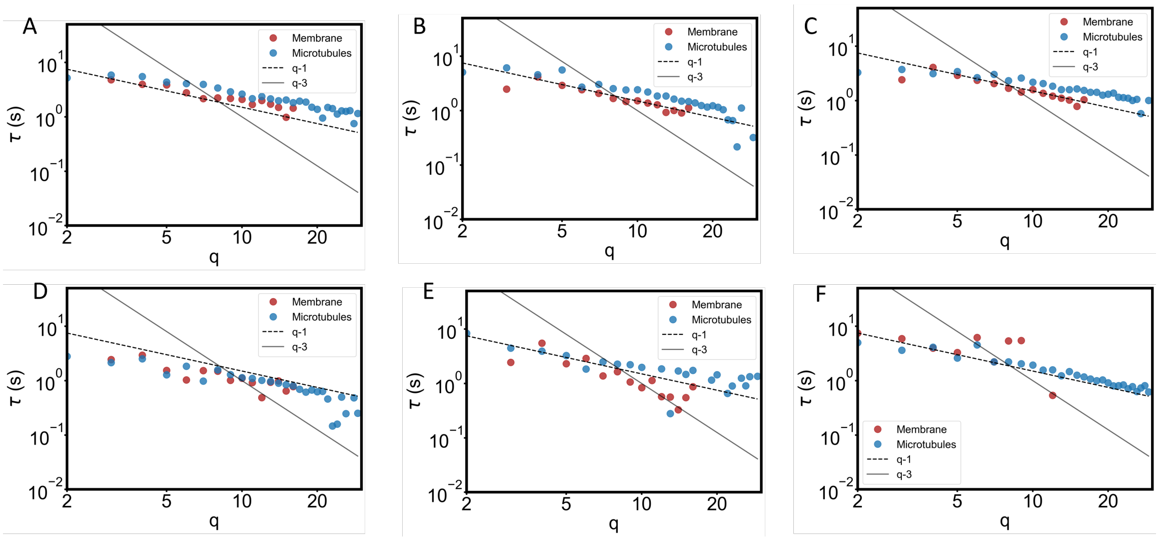

Supp.Fig. 6: Comparison between membrane and MT density correlation times in different conditions. In all cases the two quantities are synchronised and exhibit the q^{-1} scaling described. Conditions are: A-B-C: c_{MT} = 0.8 mg/ml, c_K =120 nM, c_A =1.5 μM, same day. D: same conditions, different day E: c_{MT}0.4 mg/ml, c_K =120 nM , c_A =1.5 μM. F: c_{MT}0.8 mg/ml, c_K =120 nM, c_A =4 μM

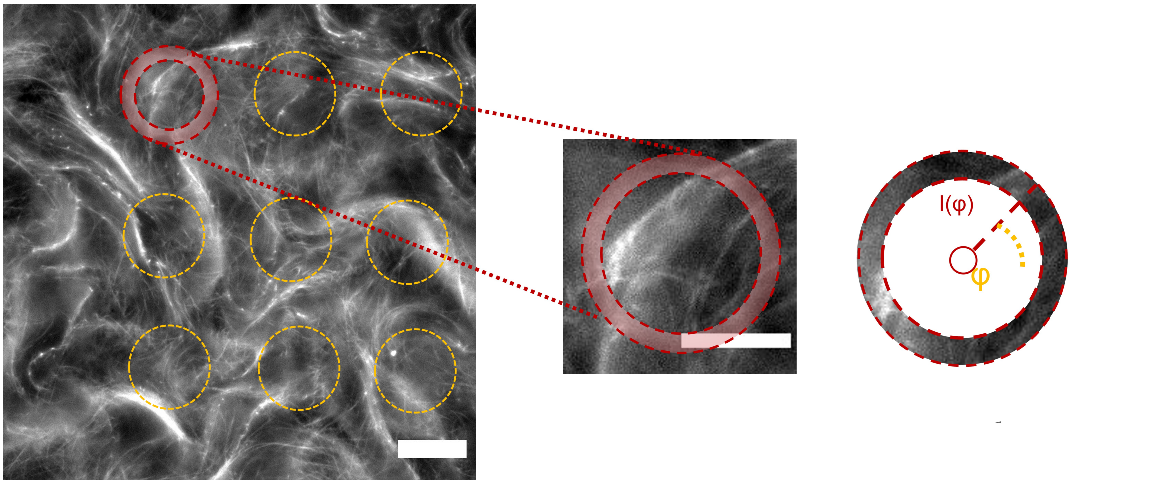

Supp.Fig. 7: Scheme of the analysis of bulk MT fluid in Fourier space. Starting from confocal planes of the bulk fluid (left), we extract the MT intensity inside circles of radius 20 μm and thickness 2 μm (center, right) and use it to compute the angular intensity as done for GUVs. This density is then converted in Fourier modes whose fluctuations are analysed. This analysis has been carried out for a single MT fluid in the same conditions as the GUV shown.

Bending elasticity is represented by the Helfrich curvature energy [8] as

$$U_{\text{bend}} = 2\kappa \oint_A \bar{c}^2 \mathrm{d}A, \tag{3}$$

A

B

C

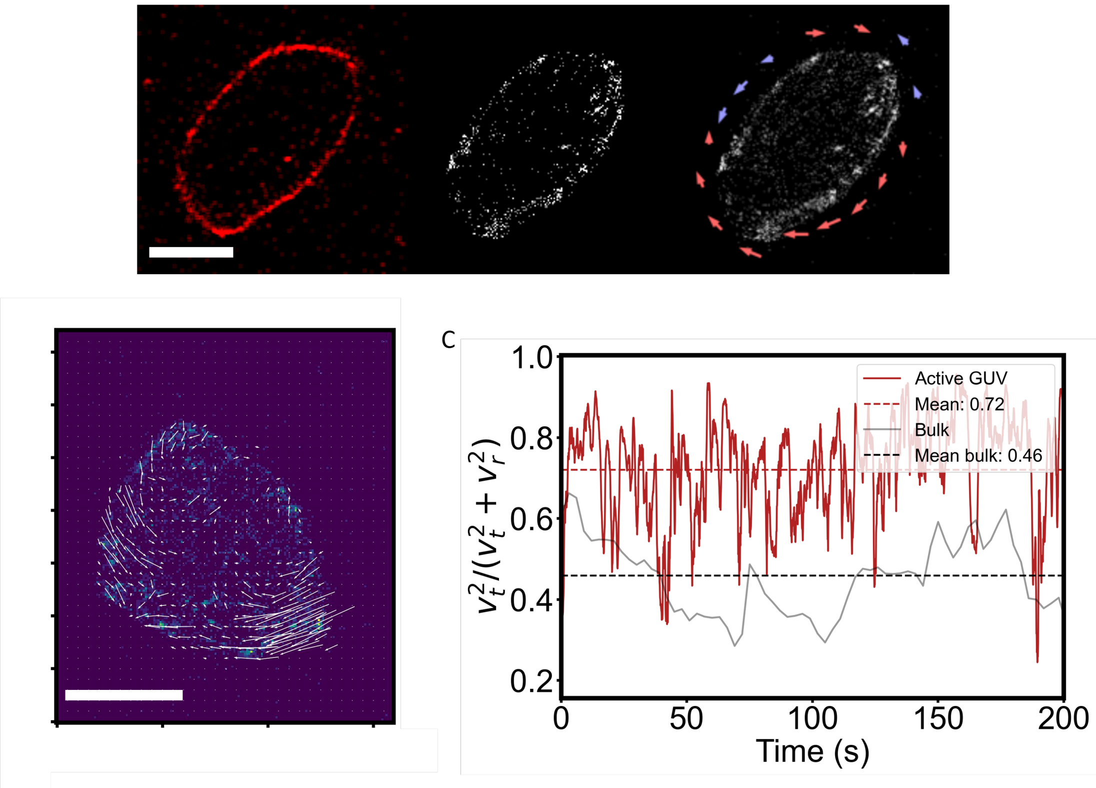

Supp.Fig. 8: A) Membrane channel, microtubule channel and extracted flow. In all cases, the membrane is identified and density and flow are computed everywhere, but then only points close to the membrane are considered. The flow is extracte dusing an optical flow algorithm and is computed only in vicinity of the membrane. Arrows' size indicate the magnitude of the speed and colors indicate domains of clockwise and counterclockwise direction of the flow. B) Example of extracted flow. C) By extracting the MT flow $\mathbf{v} = (v_x, v_y)$ inside GUVs, we can compute the ratio of the kinetic energy between the tangential speed $v_t = \mathbf{v} \cdot \mathbf{n}$ and the radial speed $v_r = \mathbf{v} \cdot \mathbf{r}$ where $\mathbf{r}$ and $\mathbf{n}$ are the radial and tangential versors ($\mathbf{n} \cdot \mathbf{r} = 0$) (shown in red for a GUV). We find that roughly ≈70% of the kinetic energy is stored in motion tangential to the membrane. In comparison, the bulk system (gray) shows as expected ≈50% (where the origin of the radial and tangential versor have been chosen at the center of the image). Scale bars are 20 μm.

where κ is the bending rigidity, $\bar{c} = (c_1 + c_2)/2$ is the local mean curvature, and A is the total membrane area. The bending energy is discretized on a triangulated network [9, 10] as

$$U_{\mathrm{bend}} = \sum_{i=1}^{N_{\mathrm{v}}} \frac{2\kappa \bar{c}_i^2 A}{N_{\mathrm{v}}} = \frac{\kappa}{2} \sum_{i=1}^{N_{\mathrm{v}}} \frac{1}{\sigma_i} \left[\mathbf{n}_i \cdot \left(\sum_{j(i)} \frac{\sigma_{ij}}{r_{ij}} \mathbf{r}_{ij} \right) \right]^2, \tag{4}$$

where $\bar{c}_i$ is the mean curvature at vertex i with an area A/N_{v}, $\mathbf{n}_i$ is a unit normal of the membrane at vertex i, and $\sigma_i = \left(\sum_{j(i)} \sigma_{ij} r_{ij} \right)/4$ is the area of dual cell of vertex i. $j(i)$ stands for all neighboring

vertices linked to the vertex i, $\sigma_{ij} = r_{ij}[\cot(\theta_1) + \cot(\theta_2)]/2$ is the length of the bond in dual lattice with angles θ_1 and θ_2 being the two angles opposite to the shared bond vector $\mathbf{r}_{ij}$. More details about discretization of the Helfrich curvature energy can be found in Refs. [9, 6, 11].

Furthermore, local triangle area conservation is imposed by a soft harmonic potential given by

$$U_{\text{loc. area}} = \frac{k_\text{l}}{2} \sum_{i=1}^{N_\text{t}} \frac{(A_i - A')^2}{A'}, \tag{5}$$

where k_l is the local-area conservation coefficient, and $A' = A/N_\text{v}$ and A_i are the desired and instantaneous local areas, respectively. The sum runs over all $N_\text{t} = 2(N_\text{v} - 2)$ triangles within the network. In most simulations, a constraint on the total vesicle volume V has been employed as

$$U_{\text{volume}} = \frac{k_\text{v} (V - V_0)^2}{2V_0}, \tag{6}$$

where k_v is the volume-constraint coefficient and V_0 is the desired total volume.

For simulations without hydrodynamics, motion of membrane vertices is modeled by the Langevin equation,

$$m_m \ddot{\mathbf{r}}_i = -\nabla_i U_{\text{tot}} - \gamma_\text{m} \dot{\mathbf{r}}_i + \sqrt{2\gamma_\text{m} k_\text{B} T} \boldsymbol{\xi}_i(t), \tag{7}$$

where m_m is the vertex mass, ∇_i is the spatial derivative at the position of vertex i, and U_{tot} is the sum of all interaction potentials. The friction coefficient γ_m mimics embedding of the membrane into a viscous fluid through the free-draining approximation. $\boldsymbol{\xi}_i(t)$ is a Gaussian random process with $\langle \boldsymbol{\xi}_i(t) \rangle = \mathbf{0}$ and $\langle \boldsymbol{\xi}_i(t) \boldsymbol{\xi}_j(t') \rangle = \mathbf{1} \delta_{ij} \delta(t - t')$ that represents membrane thermal fluctuations. The positions and velocities of all particles are integrated using the velocity-Verlet algorithm [12]. The mass is chosen small enough for inertial effects to be negligible. For several simulations with hydrodynamic interactions, the dissipative particle dynamics (DPD) method [13, 14] has been used to model fluid surroundings, see Section 3.1 for details.

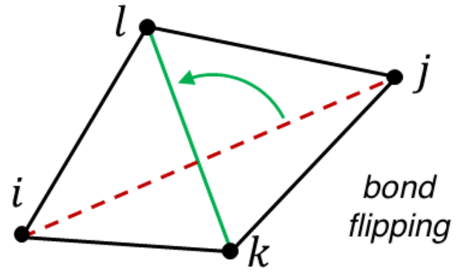

Supp.Fig. 9: Bond flipping process to represent lipid membrane fluidity. For a pair of triangles sharing a bond ij (red dashed line), this bond can be removed and a new bond kl (green line) can be created following an energy-based Monte-Carlo algorithm. The bond flipping leads to the creation of two new triangles.

The membrane model described above with a fixed network connectivity does not represent membrane fluidity where particles can diffuse within the membrane plane. In order to model membrane fluidity, bonds shared by each pair of triangles are flipped as illustrated in Fig. S9. The flipping

procedure is performed with a time frequency ν, i.e. every few time steps. During the flipping procedure, every bond in the membrane network is attempted to be flipped with a probability ψ, where the acceptance of bond flipping follows a Monte-Carlo algorithm. In the Monte-Carlo algorithm, changes in the tethering (i.e. $\Delta U_{\text{att}} + \Delta U_{\text{rep}}$) and local area (i.e. $\Delta U_{\text{loc. area}}$) energies due to attempted bond flipping are computed, and the bond flipping is accepted with a probability $\exp\left[-(\Delta U_{\text{att}} + \Delta U_{\text{rep}} + \Delta U_{\text{loc. area}})/k_B T\right]$. Note that changes in the bending and volume-constraint energies are omitted due to simplicity, as bond flipping has a negligible effect on the local membrane curvature and the total volume of a vesicle. The resulting membrane fluidity for selected parameters ν and ψ can be characterized by a 2D membrane viscosity, see Refs. [15, 7] for details.

3.1 Dissipative particle dynamics method for fluid modeling

To represent fluid surroundings, we employ the dissipative particle dynamics (DPD) method [13, 14], which is a mesoscopic hydrodynamics simulation technique. DPD is a particle-based Lagrangian method, where each particle represents a small fluid volume. DPD particles i and j interact through three types (conservative, dissipative, and random) of pairwise forces given by

$$\mathbf{F}^C(r_{ij}) = aW^C(r_{ij})\hat{\mathbf{r}}_{ij}, \tag{8}$$

$$\mathbf{F}^D(r_{ij}) = -\gamma W^D(r_{ij})(\hat{\mathbf{r}}_{ij} \cdot \mathbf{v}_{ij})\hat{\mathbf{r}}_{ij}, \tag{9}$$

$$\mathbf{F}^R(r_{ij}) = \sigma_r W^R(r_{ij})\xi_{ij}\hat{\mathbf{r}}_{ij}/\sqrt{\Delta t}, \tag{10}$$

where a, γ, and σ_r are the force amplitudes, $\mathbf{r}_{ij} = \mathbf{r}_i - \mathbf{r}_j$ is the relative position vector, $r_{ij} = |\mathbf{r}_{ij}|$, $\hat{\mathbf{r}}_{ij} = \mathbf{r}_{ij}/r_{ij}$, and $\mathbf{v}_{ij} = \mathbf{v}_i - \mathbf{v}_j$ is the velocity difference. $\xi_{ij} = \xi_{ji}$ is a symmetric Gaussian random variable with zero mean and unit variance, and Δt is the time step. All forces act within a cutoff radius r_c and vanish beyond it. The conservative force controlls fluid compressibility, while the dissipative and random forces form a thermostat, so that the DPD fluid has an isotropic temperature T. Thus, $\mathbf{F}^D$ and $\mathbf{F}^R$ are related through the fluctuation-dissipation theorem [14] as

$$\sigma_r^2 = 2\gamma k_B T, \qquad W^D(r_{ij}) = [W^R(r_{ij})]^2. \tag{11}$$

The weight functions are defined as

$$W(r_{ij}) = W^R(r_{ij}) = \begin{cases} (1 - r_{ij}/r_c)^s, & r_{ij} < r_c, \\ 0, & r_{ij} \geq r_c, \end{cases} \tag{12}$$

with an exponent s. For the conservative force, $W^C(r_{ij}) = W(r_{ij})$ with $s = 1$.

Time evolution of each DPD particle follows the Newton's second law

$$\frac{d\mathbf{r}_i}{dt} = \mathbf{v}_i, \qquad m_i \frac{d\mathbf{v}_i}{dt} = \sum_{j \neq i} \left(\mathbf{F}^C(r_{ij}) + \mathbf{F}^D(r_{ij}) + \mathbf{F}^R(r_{ij}) \right), \tag{13}$$

where m_i is the mass of particle i. Time integration is performed using the velocity-Verlet algorithm [12].

DPD parameters for the interactions between fluid particles are $a = 60 k_B T/r_c$ ($k_B T = 1$ and $r_c = 1$ in simulations), $\gamma = 22\sqrt{m k_B T}/r_c$ ($m = 1$ in all simulations), and $s = 0.15$. The number density of fluid particles is $n = 3/r_c^3$. These DPD parameters yield a fluid viscosity of $\eta = 9.5\sqrt{m k_B T}/r_c^2$. Furthermore, friction coupling of vesicle particles to fluid particles is performed using DPD parameters $a = 0$, $\gamma = 18\sqrt{m k_B T}/r_c$, and $s = 0.1$. The coupling of filaments to fluid particles assumes $a = 0$, $\gamma = 10\sqrt{m k_B T}/r_c$, and $s = 0.1$. The time step for integration is $10^{-3} r_c \sqrt{m/k_B T}$.

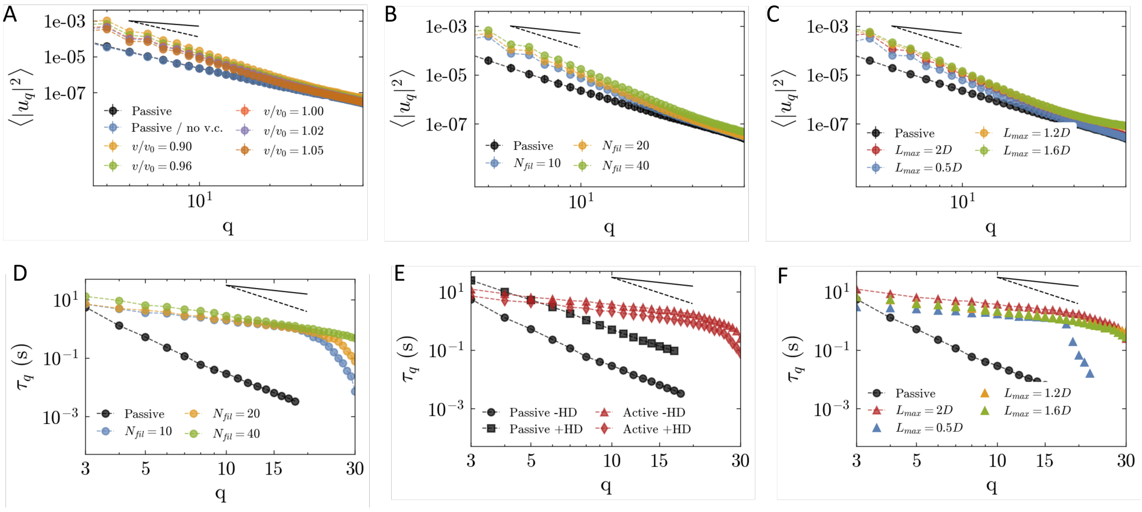

Supp.Fig. 10: A) Fluctuation spectra as the target volume V_0 is changed with respect to $v_0 = 4.05{*}R_0^3$ and for the cases without volume constraint applied. Passive vesicle spectrum is also shown. B) Fluctuation spectra as the number of growing filaments is changed. Passive vesicle spectrum is also shown. C) Fluctuation spectra as the maximum length of growing filaments is changed. Passive vesicle spectrum is also shown. D) Correlation times as the number of growing filaments is changed. Passive vesicle spectrum is also shown. E) Correlation times in the active and passive case with and without hydrodynamic interactions (HD). As expected the scaling for a passive vesicle changes from q^{-4} to q^{-3} as HD is introduced, while in the active case, the presence of HD is irrelevant for the scaling. F) Correlation times as the maximum length of growing filaments is changed. $D = 2R_0$. Passive vesicle times are also shown. The q^{-1} and q^{-3} scalings are also shown in all plots by solid and dashed lines, respectively.

3.2 Growing semiflexible filaments

Growing semiflexible filaments are modeled by bead-spring chains composed of N_b beads. The beads are subject to bond and bending potentials; however, no repulsion between beads is applied. The bond energy controls the length of the filaments, and is represented by a harmonic bond potential

$$U_\mathrm{s} = k_\mathrm{s} \sum_{i=1}^{N_\mathrm{b}-1} (|\mathbf{r}_i| - l_\mathrm{s}(t))^2, \tag{14}$$

where k_s is the spring constant, $l_\mathrm{s}(t)$ is the time-dependent bond length, and $\mathbf{r}_i$ is the bond vector from monomer i to $i+1$.

The curvature (or bending) energy is given by

$$U_\mathrm{b} = \frac{\kappa_f}{l_\mathrm{s}(t)} \sum_{i=2}^{N_\mathrm{b}-1} (\theta_i - \theta_0)^2, \tag{15}$$

where κ_f is the 2D bending rigidity of filaments, θ_i is the angle formed by consecutive beads $i-1$, i, and $i+1$, and θ_0 is the preferred angle. In all simulations, $\theta_0 = \pi$, corresponding to a straight

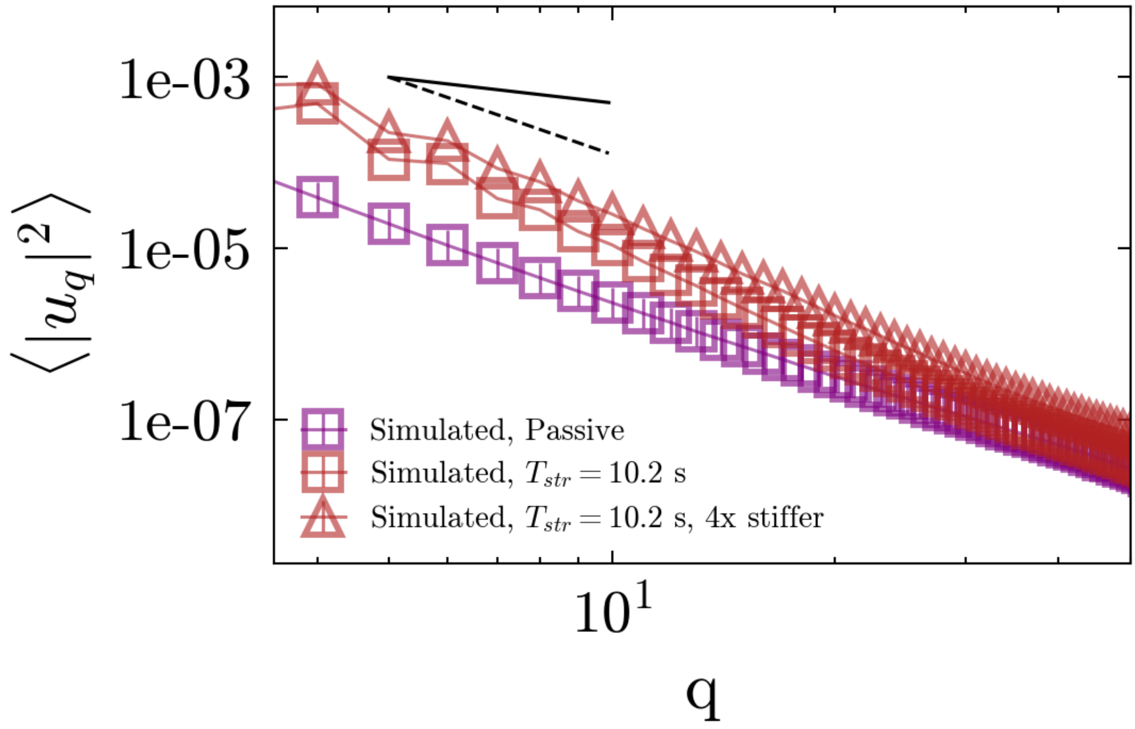

Supp.Fig. 11: Fluctuation spectra for a passive (purple) and two active (red) vesicles ($T_{str} = 10.2$ s) for two different filament bending rigidities $\kappa_f = 160 k_B T R_0$ (triangles) and $\kappa_f = 640 k_B T R_0$ (squares). Data show that the fluctuation amplitudes increase for high mode numbers with increasing filament rigidity.

filament in equilibrium. Note that the division by $l_s(t)$ in Eq. (15) ensures a constant bending rigidity κ_f of filaments, independently of their length.

The active vesicle always contains N_{fil} growing filaments. Filament growth is implemented trough the time-dependent bond length $l_s(t)$, which has a cycle of three phases: (i) growth delay, (ii) growth, and (iii) shrinkage. Initially, each filament is generated with a randomly distributed orientation and $l_s = r_{\text{min}}$ the minimum bond length. The center of mass of each filament is offset by d_{offset} from the center of mass of the vesicle in a random direction, so that filaments are not always placed centrally within the vesicle. First, each filament is assigned a growth delay time t_{delay} during which $l_s = r_{\text{min}}$ remains unchanged, i.e. the filament does not grow. t_{delay} is drawn from a uniform distribution $U[\bar{t}_{\text{delay}} - \Delta t_{\text{delay}}, \bar{t}_{\text{delay}} + \Delta t_{\text{delay}}]$, where $\bar{t}_{\text{delay}}$ is the average delay time and Δt_{delay} defines growth delay range. Non-constant delay times ensure decorrelation of growth of different filaments. After the growth delay time has passed, the filament growth starts, such that l_s increases linearly from r_{min} to r_{max} (the maximum bond length) during the growth time t_{growth}. When the growth is over, the filament shrinks, such that l_s decreases linearly from r_{max} to r_{min} during the shrinkage time t_{shrink}. When this cycle of three phases is finished, the filament is deleted and a new filament with a random orientation is generated to follow a new growth cycle.

Excluded-volume interactions between the filament beads and vesicle particles are implemented

through a repulsive part of the 12-6 Lennard-Jones (LJ) potential given by

$$U_{\mathrm{LJ}}(r) = \begin{cases} \epsilon_{\mathrm{LJ}} \left[\left(\frac{r_{\mathrm{LJ}}}{r}\right)^{12} - 2\left(\frac{r_{\mathrm{LJ}}}{r}\right)^{6} \right] & \text{if } r < \sqrt[6]{2} r_{\mathrm{LJ}} \\ 0 & \text{if } r \geq \sqrt[6]{2} r_{\mathrm{LJ}} \end{cases}, \tag{16}$$

where ϵ_{LJ} is the strength of the LJ potential and r_{LJ} is a characteristic length of repulsion. For simplicity, there is no repulsion between beads within each filament and between different filaments. This means that filaments are allowed to cross each other.

For simulations without hydrodynamic interactions, dynamics of growing semiflexible filaments is governed by the Langevin equation (7) with the bead mass m_f and friction γ_f. To have a constant friction per unit length, we set $\gamma_f = l_s \gamma'_f / r_{\mathrm{min}}$, where γ'_f is a constant friction coefficient. For simulations with hydrodynamics, filament beads are coupled to the DPD fluid through the dissipative and random forces, similar to the vesicle-fluid coupling. The conservative force for the filament-fluid coupling is turned off.

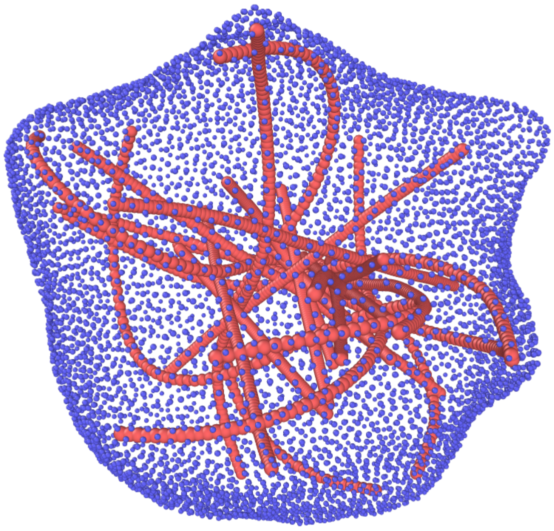

Supp.Fig. 12: Simulation snapshot, where blue particles represent the vesicle and red particles form growing filaments.

3.3 Simulation setup and extracted quantities

Simulation setup consists of a vesicle with $N_{\mathrm{v}} = 6536$ and N_{fil} growing filaments each with $N_{\mathrm{b}} = 101$, see a snapshot in Fig. S12. Table S1 summarizes simulation parameters used. A length scale is set by the vesicle radius R_0 in equilibrium, the energy scale by $k_{\mathrm{B}}T = 1$, and the time scale is defined as $\tau = \eta R_0^3/\kappa$, where η is the dynamic viscosity ($\eta = 9.5$ was selected for simulations). The simulation domain is set to $5R_0 \times 5R_0 \times 5R_0$. To restrict spontaneous motion of the vesicle in the simulation domain, its center of mass $\mathbf{x}_{\mathrm{CM}}$ is tethered to the coordinate origin, using a harmonic potential $U_{\mathrm{tether}} = k_{\mathrm{tether}} x_{\mathrm{CM}}^2$.

For Langevin simulations without hydrodynamics, friction on each vesicle particle is assumed to be $\gamma_{\mathrm{m}} = 6\pi\eta R_0/N_{\mathrm{v}}$, while friction on each filament bead $\gamma'_{\mathrm{f}} = 3\pi\eta L_f/N_{\mathrm{b}} = 3\pi\eta r_{\mathrm{min}}$, where L_f is

the minimum filament length. Note that the reduced volume of the vesicle $V_0/(4/3\pi R_0) = 0.97$ is chosen slightly below unity, such that the vesicle remains nearly spherical and does not develop a measurable tension in equilibrium. The time step in simulations is $\Delta t = 2.63 \times 10^{-7}\tau$. The total simulation time corresponds to approximately 13τ.

Table 1: Simulation parameters.

Parameter	Model units	Physical units
	Principal properties	
vesicle radius in equil. R_0	20	20 μm
therm. energy $k_\mathrm{B}T$	1.0	4.05×10^{-21} J
dynamic viscosity η	9.5	10^{-3} Pa $\cdot$ s
time scale, $\tau = \eta R_0^3/\kappa$	3800	192.9 s
	Vesicle properties	
no. of vertices N_v	6536	6536
bending rigidity κ	$20 k_\mathrm{B}T$	8.1×10^{-20} J
avg. bond length l_b	$4R_0\sqrt{\frac{\pi}{N_\mathrm{t}\sqrt{3}}}$	1.18 μm
bond stiffness k_b	$80 k_\mathrm{B}T$	3.24×10^{-19} J
min. bond length l_min	$0.6 l_\mathrm{b}$	0.71 μm
potential cutoff length l_{c1}	$0.8 l_\mathrm{b}$	0.94 μm
potential cutoff length l_{c0}	$1.2 l_\mathrm{b}$	1.41 μm
max. bond length l_max	$1.4 l_\mathrm{b}$	1.65 μm
equil. area A	$4\pi R_0^2$	7.85×10^{-9} m^2
loc. triangle area A'	A/N_v	6.01×10^{-13} m^2
target volume V_0	$4.05 R_0^3$	6.33×10^{-14} m^3
local area stiffness k_l	4.0×10^4 $k_\mathrm{B}T/R_0^2$	2.3×10^{-7} J/m^2
volume stiffness k_v	8.0×10^5 $k_\mathrm{B}T/R_0^3$	0.21 J/m^3
translat. fric. on mem. vertex γ_m	$6\pi\eta R_0/N_\mathrm{v}$	7.21×10^{-11} J $\cdot$ s $\cdot$ m^{-2}
flipping frequency ν	3.8×10^5 τ^{-1}	2.0×10^3 s^{-1}
flipping probability ψ	0.3	0.3
tethering spring constant k_tether	2.0×10^6 $k_\mathrm{B}T/R_0^2$	1.15×10^{-5} J/m^2
	Filament properties	
number of filaments N_fil	$0 - 40$	$0 - 40$
number of beads N_b	101	101
spring constant k_s	4.0×10^6 $k_\mathrm{B}T/R_0^2$	2.3×10^{-5} J/m^2
min. spring length r_min	$0.01 R_0$	0.25 μm
max. spring length r_max	$0.025 R_0 - 0.08 R_0$	$0.625 - 2$ μm
mean delay time $\bar{t}_\mathrm{delay}$	0.026τ	5 s
delay deviation time Δt_delay	0.013τ	2.5 s
growth time t_growth	$0.0026\tau - 0.11\tau$	$0.5 - 21$ s
shrinkage time t_shrink	0.0013τ	0.25 s
bending rigidity κ_f	160 $k_\mathrm{B}TR_0$	1.62×10^{-23} Jm
LJ potential energy ϵ_LJ	$1 k_\mathrm{B}T$	4.05×10^{-21} J
LJ repulsion length r_LJ	$0.075 R_0$	1.875 μm
translat. fric. γ_f'	$3\pi\eta r_\mathrm{min}$	2.36×10^{-9} J $\cdot$ s $\cdot$ m^{-2}

3.4 Computation of spectra and correlation times

The membrane fluctuation spectrum is computed from simulated data using the radial positions of the membrane $R(\phi_m, t_i)$ at an equatorial plane for polar angles $\phi_m = 2\pi m/N_{ang}$ ($m = 0 \ldots N_{ang} - 1$, $N_{ang} = 200$) and different times t_i. The values of $R(\phi_m, t_i)$ at the vesicle cross-section are obtained by local surface interpolation within the section plane. Then, the vesicle contour $R(\phi_m, t_i)$ is fast Fourier transformed for each time t_i to obtain $u_q(t_i)$ coefficients, which are normalized by the vesicle radius R_0 and used to compute the fluctuation spectrum. Furthermore, temporal correlation

functions are computed for each u_q coefficient, and used to extract the values of the correlation time τ_q by exponential fitting. The obtained values are then converted in physical units using the length scale R_0, the energy scale $k_{\mathrm{B}}T$, and the time scale τ.

3.5 Computation of the active force and the corresponding τ_q^a values

Active force on each vesicle vertex is computed as the force exerted by growing filaments on the membrane. Absolute values of the active force on membrane vertices are computed during simulations and saved for different times t_i. To obtain a cross-sectional cut from active forces distributed on the vesicle, we use force values at the membrane vertices located within an equatorial section of thickness $h = 0.1R_0$. These membrane vertices are divided into 100 polar bins, in which the active forces are summed up. Then, the active force contour is fast Fourier transformed for each time t_i to obtain $u_q^a(t_i)$ coefficients. Finally, temporal correlation functions are computed for each u_q^a coefficient, and used to extract the values of the correlation time τ_q^a by exponential fitting.

3.6 Computation of the tension

Membrane tension in simulations is computed from the local virial contribution to the stress. Thus, local tension is computed for each membrane particle and then, averaged over the whole vesicle (i.e., over all membrane vertices). For a passive vesicle, the computed membrane tension is $\sigma \approx 10^{-8}\ N/m$, which is somewhat smaller than that estimated in experiments but still provides a good fit in the bending dominated case.

3.7 Computation of the crossover mode

To compute the crossover mode for various κ, the first 5 points (i.e., those at low mode numbers q) are fitted in log-scale with a line, i.e. as $log(\tau_q) = -m\log(q) + c$, with parameters m and c. The crossover mode is defined by the first mode with $q > 4$, for which $|log(\tau_q) + m\log(q) - c| > 1$ (in simulation units).

References

[1] Faizi, H. A., Reeves, C. J., Georgiev, V. N., Vlahovska, P. M. & Dimova, R. Fluctuation spectroscopy of giant unilamellar vesicles using confocal and phase contrast microscopy. *Soft Matter* **16**, 8996–9001 (2020).

[2] Gracià, R. S., Bezlyepkina, N., Knorr, R. L., Lipowsky, R. & Dimova, R. Effect of cholesterol on the rigidity of saturated and unsaturated membranes: Fluctuation and electrodeformation analysis of giant vesicles. *Soft Matter* **6**, 1472–1482 (2010).

[3] Battle, C. *et al.* Broken detailed balance at mesoscopic scales in active biological systems. *Science* **352**, 604–607 (2016).

[4] Kokot, G., Faizi, H. A., Pradillo, G. E., Snezhko, A. & Vlahovska, P. M. Spontaneous self-propulsion and nonequilibrium shape fluctuations of a droplet enclosing active particles. *Communications Physics* **5**, 91 (2022).

[5] Kroll, D. M. & Gompper, G. The conformation of fluid membranes: Monte Carlo simulations. *Science* **255**, 968–971 (1992).

[6] Gompper, G. & Kroll, D. M. Triangulated-surface models of fluctuating membranes. In Nelson, D. R., Piran, T. & Weinberg, S. (eds.) *Statistical mechanics of membranes and surfaces*, 359–426 (World Scientific, Singapore, 2004), 2nd edn.

[7] Noguchi, H. & Gompper, G. Dynamics of fluid vesicles in shear flow: effect of the membrane viscosity and thermal fluctuations. *Phys. Rev. E* **72**, 011901 (2005).

[8] Helfrich, W. Elastic properties of lipid bilayers: theory and possible experiments. *Z. Naturforsch.* **28**, 693–703 (1973).

[9] Gompper, G. & Kroll, D. M. Random surface discretizations and the renormalization of the bending rigidity. *J. Phys. I France* **6**, 1305–1320 (1996).

[10] Gompper, G. & Kroll, D. M. Network models of fluid, hexatic and polymerized membranes. *J. Phys.: Condens. Matter* **9**, 8795–8834 (1997).

[11] Guckenberger, A. & Gekle, S. Theory and algorithms to compute Helfrich bending forces: a review. *J. Phys.: Condens. Matter* **29**, 203001 (2017).

[12] Allen, M. P. & Tildesley, D. J. *Computer simulation of liquids* (Clarendon Press, New York, 1991).

[13] Hoogerbrugge, P. J. & Koelman, J. M. V. A. Simulating microscopic hydrodynamic phenomena with dissipative particle dynamics. *Europhys. Lett.* **19**, 155–160 (1992).

[14] Español, P. & Warren, P. Statistical mechanics of dissipative particle dynamics. *Europhys. Lett.* **30**, 191–196 (1995).

[15] Noguchi, H. & Gompper, G. Fluid vesicles with viscous membranes in shear flow. *Phys. Rev. Lett.* **93**, 258102 (2004).
